# Mitii™ ABI: study protocol of a randomised controlled trial of a web-based multi-modal training program for children and adolescents with an Acquired Brain Injury (ABI)

**DOI:** 10.1186/s12883-015-0381-6

**Published:** 2015-08-19

**Authors:** Roslyn N. Boyd, Emmah Baque, Adina Piovesana, Stephanie Ross, Jenny Ziviani, Leanne Sakzewski, Lee Barber, Owen Lloyd, Lynne McKinlay, Koa Whittingham, Anthony C. Smith, Stephen Rose, Simona Fiori, Ross Cunnington, Robert Ware, Melinda Lewis, Tracy A. Comans, Paul A. Scuffham

**Affiliations:** 1 grid.1003.20000000093207537Queensland Cerebral Palsy and Rehabilitation Research Centre, School of Medicine, The University of Queensland, Brisbane, Queensland Australia; 2 Children’s Allied Health Research, Children’s Health Queensland, Brisbane, Queensland Australia; 3 grid.1003.20000000093207537School of Health and Rehabilitation Sciences, The University of Queensland, Brisbane, Queensland Australia; 4 grid.1003.20000000093207537Centre for Online Health, The University of Queensland, Brisbane, Australia; 5 grid.1003.20000000093207537CSIRO, ICT – Australian e-Health Research Centre, Royal Brisbane and Women’s Hospital Centre for Clinical Research, The University of Queensland, Brisbane, Queensland Australia; 6 Department of Developmental Neuroscience, IRCCS Stella Maris, Pisa, Italy; 7 grid.1003.20000000093207537Queensland Brain Institute, The University of Queensland, Brisbane, Queensland Australia; 8 grid.1003.20000000093207537School of Psychology, The University of Queensland, Brisbane, Queensland Australia; 9 grid.1003.20000000093207537Queensland Children’s Medical Research Institute, The University of Queensland, Brisbane, Queensland Australia; 10 grid.1003.20000000093207537School of Population Health, The University of Queensland, Brisbane, Queensland Australia; 11 grid.1022.10000000404375432Griffith Health Institute and School of Medicine, Griffith University, Brisbane, Queensland Australia

**Keywords:** Acquired Brain Injury, Children, Physical Activity, Virtual Reality, Motor Processing, Protocol, Executive Function, Randomised Controlled Trial

## Abstract

**Background:**

Acquired brain injury (ABI) refers to multiple disabilities arising from damage to the brain acquired after birth. Children with an ABI may experience physical, cognitive, social and emotional-behavioural impairments which can impact their ability to participate in activities of daily living (ADL). Recent developments in technology have led to the emergence of internet-delivered therapy programs. “Move it to improve it” (Mitii™) is a web-based multi-modal therapy that comprises upper limb (UL) and cognitive training within the context of meaningful physical activity. The proposed study aims to compare the efficacy of Mitii™ to usual care to improve ADL motor and processing skills, gross motor capacity, UL and executive functioning in a randomised waitlist controlled trial.

**Methods/Design:**

Sixty independently ambulant children (30 in each group) at least 12 months post ABI will be recruited to participate in this trial. Children will be matched in pairs at baseline and randomly allocated to receive either 20 weeks of Mitii™ training (30 min per day, six days a week, with a potential total dose of 60 h) immediately, or be waitlisted for 20 weeks. Outcomes will be assessed at baseline, immediately post-intervention and at 20 weeks post-intervention. The primary outcomes will be the Assessment of Motor and Process Skills and 30 s repetition maximum of functional strength exercises (sit-to-stand, step-ups and half kneel to stand). Measures of body structure and functions, activity, participation and quality of life will assess the efficacy of Mitii™ across all domains of the International Classification of Functioning, Disability and Health framework. A subset of children will undertake three tesla (3T) magnetic resonance imaging scans to evaluate functional neurovascular changes, structural imaging, diffusion imaging and resting state functional connectivity before and after intervention.

**Discussion:**

Mitii™ provides an alternative approach to deliver intensive therapy for children with an ABI in the convenience of the home environment. If Mitii™ is found to be effective, it may offer an accessible and inexpensive intervention option to increase therapy dose.

**Trial Registration:**

ANZCTR12613000403730

**Electronic supplementary material:**

The online version of this article (doi:10.1186/s12883-015-0381-6) contains supplementary material, which is available to authorized users.

## Background

### Introduction

Acquired brain injury (ABI) is an umbrella term that refers to any insult to the brain in the post-neonatal period [1]. Generally, the minimum age for an ABI is 28 days post full-term birth, allowing for a recognised event with brain damaging potential (that is not likely to be attributed to any events in the intrauterine environment) in a previously well infant [2]. Common causes of childhood ABI include traumatic brain injury (TBI), stroke, infection, cerebral tumour, cerebral hypoxia or anoxia and encephalitis [1]. Recent advances in medical response have led to decreased mortality rates following brain injury (including TBI, brain tumour and stroke), with more individuals living with the sequelae of ABI [3]. Over 600,000 Australians have a brain injury with Queensland having the highest prevalence (2.5 % compared to 1.8 % for the rest of Australia) [1]. As many as two out of three of these people acquired their brain injury before 25 years of age with the majority sustaining a TBI [4]. The estimated lifetime cost per incident case is AUD$ 4.8 million for moderate and severe TBI of which 19 % is borne directly by state governments [3]. These figures include financial costs involved with health care, equipment and modifications, long-term care, productivity losses and burden of disease costs. This highlights the importance of developing effective treatments and interventions beyond acute medical care to enhance post-injury outcomes and minimise the economic impact of ABI at an individual and government level.

Many children with an ABI experience persistent academic, occupational, physical, emotional-behavioural and cognitive impairments leading to reduced quality of life (QOL) [5–7]. Behavioural deficits, such as anxiety and depression may also deteriorate over time because of damage to specific areas of the brain and increased aggression and hyperactivity may not manifest until several years after injury [8, 9]. All of these impairments impact on a child’s ability to successfully participate in therapeutic interventions post discharge. Secondary psychosocial factors including family material and social resources, social disadvantage, stressors, parent distress and general family functioning may also emerge requiring long-term management by a multi-disciplinary team [10].

Although motor outcomes recover more quickly than cognitive skills after ABI, ongoing physical impairments may remain. Physical impairment is closely related to brain injury severity, whereby greater brain injury severity predicts poorer physical (and cognitive) outcomes [5–7]. Common physical impairments following ABI include changes in muscle tone, impaired balance and sensation, reduced strength and coordination [11, 12]. The Australian Bureau of Statistics 2003 Survey of Disability, Ageing and Carers found that nearly all of the 20,000 children (90 %) who were identified as having an ABI as a main or associated diagnosis were reported to have “severe or profound core activity limitations” [4]. Motor difficulties impact a child’s ability to engage in activities of daily living (ADL) such as getting dressed and walking. Recovery of functional abilities and support for ongoing acquisition of developmental skills is a key goal of inpatient and outpatient rehabilitation so children can return to participating in age appropriate activities.

Cognitive impairment is a common consequence of ABI [5, 13–15], with children experiencing difficulties with memory, attention, concentration, executive dysfunction and a potential decline in intellectual ability over time [13, 14]. Previous studies have reported that it is often executive control over cognitive processes, rather than specific processes themselves (e.g. memory) that is impaired and requires rehabilitation [14–16]. Often such cognitive deficits following an ABI can lead to poor school attainment, behavioural issues and impact personal relations and employment opportunities [14].

### Upper Limb (UL) interventions

Even though ABI is one of the leading causes of long-term disability in children and young adults, there is limited evidence to guide choice of therapy. There is preliminary low level evidence for bimanual training (BIM), forced used therapy and modified-Constraint Induced Movement Therapy (mCIMT) to improve UL function in children with an ABI [17–21]. Forced use therapy was compared to a control group in a randomised controlled trial (RCT) for 25 children with ABI aged one to eight years [19]. Forced use involved continuous casting of the unimpaired UL for one month with continuation of regular occupational therapy and physiotherapy. Forced use therapy led to improved fine motor skills, as measured on the Peabody Developmental Motor Scales, compared to the control group [19].

A recent RCT compared two intensive UL approaches in 33 children with unilateral cerebral palsy (CP) and 14 children with non-progressive hemiplegia (stroke and TBI) [20]. Children either received 60 h of mCIMT and 20 h of BIM or 80 h of BIM training over four weeks. Both interventions led to a significant improvement in hand motor function, however mCIMT yielded greater changes in unimanual capacity (as measured by the Melbourne Assessment of Unilateral Upper Limb Function, MUUL) [20]. Data for the subgroup of children with an ABI were not reported separately due to the small numbers, therefore it is unclear the extent of treatment effect in children with an ABI [20]. Three studies have investigated the use of mCIMT for children with an ABI using case series design [18, 17, 21]. A pilot study investigated the effectiveness of 40 h of mCIMT over four weeks in eight children aged 6 to 15 years at least one year after arterial ischaemic stroke. After mCIMT there were no improvements in sensorimotor function or quality of UL movement (MUUL) [21]. All children reportedly improved occupational performance goals as measured on the Canadian Occupational Performance Measure (COPM) and Goal Attainment Scaling (GAS) however, no data were provided [21]. A second study of 10 children aged 8 to 12 years with TBI received mCIMT for three hours per day, seven days a week over a 10 week period (total dose of 210 h) and demonstrated gains in dissociated movements and grasp on the Quality of Upper Extremity Skills Test [18]. A final study investigated mCIMT provided for six hours per day, five days per week over two weeks (total dose of 60 h) for seven children with an ABI aged 7 to 17 years, demonstrating gains in the amount and quality of use of the impaired UL [17]. It is difficult to compare results across trials as they targeted different age groups, types of ABI and used vastly different treatment protocols (total doses ranged from 40 to 210 h). It appears, however, that at least 60 h of intensive training was required to drive changes in UL motor outcomes. This has implications for current therapeutic interventions in children with an ABI.

Limited evidence is available for other therapeutic interventions such as repetitive Transcranial Magnetic Stimulation (rTMS), motor imagery and mirror therapy to improve UL function in children with an ABI [22]. Repetitive Transcranial Magnetic Simulation involves non-invasive brain stimulation, delivered by a pulsed magnetic field has been investigated in a small RCT of 10 children after stroke [23]. Improved grip strength was evident after eight days of daily rTMS [23]. The majority of evidence for motor imagery and mirror therapy has been in adult ABI populations and although evidence is promising, the methodological quality is limited [22].

### Physical activity and functional strength interventions

Gross motor capacity refers to what movements (which usually incorporate large muscle groups) a person can do in a standardised environment [24]. There is preliminary evidence to support the use of home-based exercise programs to improve gross motor capacity in children and adolescents with an ABI [25, 26]. One RCT has evaluated the immediate and short term effects of a home-based exercise program to improve functional capacity for children aged 7 to 13 years with TBI (n = 10) and CP (n = 10) [25]. The intervention consisted of six weeks of task-oriented exercises including sit-to-stands and front and sideways step-ups. Children in the intervention group improved on motor and balance performance as measured by the Timed Up and Go (TUG) test and Functional Reach Test after six weeks of training [25]. The results for the TBI group should however, be interpreted cautiously as it is unknown whether results reached clinical significance and whether a larger sample size would have demonstrated greater changes.

Another non-randomised, within subject controlled study investigated the effects of a supervised home-based motor training program utilising sit-to-stands and step-up exercises [26]. Nineteen children aged 5 to 15 years at least one year post ABI with a Glasgow Coma Scale (GCS) equal to or less than eight at the time of injury were included [26]. Children were evaluated at baseline, after the control period (four weeks of no intervention) and then four weeks post-intervention [26]. Within-group comparisons showed significant improvements in walking speed, walking distance and balance items on the Bruininks-Oseretsky Test of Motor Proficiency (BOT-MP) [26]. These studies provide preliminary evidence for the use of home-based exercise program to improve gross motor capacity in children with an ABI.

Habitual Physical Activity (HPA) can be defined as any bodily movement produced by skeletal muscles resulting in energy expenditure [27]. Fitness level is an important health indicator [28] and participation in regular physical activity is associated with improved self-efficacy to perform ADL in children with a physical disability [29]. Understanding HPA patterns will provide insight into both health status and functional ability for these children. Habitual physical activity in children and adolescents can be measured subjectively using self-report questionnaires, activity logs and diaries as well as objectively using direct observation, doubly labelled water, heart rate monitoring, accelerometers and pedometers [30]. Accelerometers have become the most widely used objective measure of physical activity as indirect calorimetry and direction observation are not feasible when measuring HPA in free-living individuals [31]. The amount of HPA in which children and adolescents with an ABI participate has only been measured subjectively using parental or self-report questionnaires and may be prone to bias (e.g. incidental or unstructured activity may be missed).

The World Health Organisation (WHO) recommends that children and adolescents aged 5–17 years should accumulate at least 60 min of moderate to vigorous physical activity [28] and the new Australian Physical Activity and Sedentary Behaviour Guidelines also suggest limiting screen time to less than two hours per day [32, 33]. Despite the health benefits and recommended guidelines, Australian children at least one year post-ABI are 25-75 % less likely to participate at school, home and in the community compared to their peers (as reported by caregivers) and are at a higher risk of obesity and co-morbidities such as hypertension [34, 35]. Despite these concerning reports, no studies have evaluated the effect of any intervention on HPA objective outcomes in children with an ABI.

### Cognitive interventions

Evidence for cognitive rehabilitation following paediatric ABI has produced mixed results [36, 37]. Research for children with an ABI has found that memory and attention respond better to targeted interventions than cognitive functions (e.g. information processing speed) [14, 36, 38]. Cognitive rehabilitation techniques have been demonstrated to be effective in adult populations however, there is a paucity of research in the paediatric population [14, 37]. Adult cognitive rehabilitation provides rationales for effective strategies but does not consider the complexities of a developing brain [14]. Evidence for attention rehabilitation in a paediatric population has shown promise for attention training and the use of compensatory strategies to remediate attention difficulties following an ABI [14]. There is limited evidence to support to use of specific training strategies for aspects of memory and more substantial evidence for the use of compensatory and external strategies to improve day to day memory functioning [14]. Executive functioning is often thought to underlie and control cognitive processes, thus requiring rehabilitation rather than the specific cognitive domains (e.g. attention and memory) [15]. Studies so far have found efficacy for cognitive and behavioural interventions, however these interventions often require intensive support and specific generalisation training [14]. Most research in executive functioning interventions focuses on a single area of executive functioning e.g. problem solving [13, 14]. There is a need for research grounded within a paediatric developmental model of executive functioning in order to inform evidence based interventions.

### Virtual Reality (VR) technologies

Internet-delivered virtual reality (VR) technologies have emerged as a feasible way to deliver intensive therapy to children and adolescents with an ABI [39]. They have the capacity to provide tailored therapy to children who may be limited by resources in their demographically-isolated communities. Virtual reality systems utilise hardware and software options to create interactive simulations that embed and engage the user in realistic environments. Virtual reality systems relevant to paediatric rehabilitation range from rehabilitation-specific technologies (e.g. GestureTek Interactive Rehabilitation Exercise System) through to off-the-shelf gaming systems (e.g. Nintendo Wii Sports and Wii Fit). In general, commercially available systems offer a wider variety of games however; their ability to manipulate and track therapeutically relevant system variables (i.e. the ability to adjust the number of repetitions, bodily movements that are recognised, speed of stimulus, complexity and grading of the challenge in games) are limited [40]. In contrast, rehabilitation specific technologies offer a wider variety of system variables that can be manipulated but have a limited number of game options available [40].

Preliminary evidence for the use of VR technologies to improve UL and lower limb (LL) activity outcomes for children with an ABI is emerging. In a pilot study, Wille et al. [41] used the rehabilitation specific Paediatric Intensive Therapy System, three sessions a week for three weeks, to improve hand function in five children with congenital and acquired brain injuries. A systematic review of VR UL rehabilitation among an adult ABI population reported moderate evidence for the use of VR to improve general UL function (including motor function, range of motion [ROM] and speed and dexterity) [42]. The majority of these studies have used small-sample or case study designs and did not adequately report specific brain injury characteristics.

A limited number of case studies to date have explored the effects of VR interventions to improve balance abilities in children and adolescents with an ABI. Two case studies with small samples (n = 3; n = 2) investigated the use of the Nintendo Wii to improve dynamic balance over a period of 3–4 weeks (total dose 2.5-6 h) [43, 44] and another single case study used both the X-box Kinect and Nintendo Wii to improve balance abilities in a 10 year old male with a non-TBI over 4 weeks (total combined dose of 7.5 h) [45]. All studies demonstrated improved balance abilities post-intervention however, two studies included participants less than a year post-injury and it was unknown whether improvements were due to the intervention or natural course of recovery from the ABI [44, 45]. Although, there are no known VR interventions targeted to improve functional capacity in children with an ABI a systematic review highlighted substantial evidence for the use of VR interventions over standard therapy in adults post-stroke [46]. Interventions using either rehabilitation specific or commercially available VR systems were significantly more effective when compared to standard therapy to improve functional capacity outcomes in this patient group. Further investigations into the use of VR systems for UL and LL rehabilitation in children with an ABI are warranted and studies should also consider possible improvements in cognitive function and societal participation.

### “Move it to improve it” (Mitii™)

“Move it to improve it” (Mitii™) is an internet-based multi-modal therapy program which comprises UL, physical and cognitive training. The program was developed through collaboration between The Helene Elsass Center, a private software development company (Headfitted; Aarhus, Denmark) and the University of Copenhagen [47]. The program design was underpinned by knowledge that training must be intense, incrementally challenging, motivating and long-lasting in order to drive neuroplastic changes in the brain [48]. The original version of Mitii™ used green tracking bands that were worn around the hands, knees or head and tracked by a web-camera attached to the computer [47]. The second generation Mitii™ is accessed in the individual’s home via the internet and requires an internet-connected computer and Microsoft Kinect® (2^nd^ generation, Redmond, Washington, USA) to track body movements.

Occupational therapists, physiotherapists and neuropsychologists act as remote “virtual trainers” and set up individual programs for each child. The therapists receive feedback about the participant’s training performance (e.g. speed of performance and percentage of answers correct) and adjustments to the program can be made to provide incremental challenge. The feasibility of the Mitii™ program has been confirmed in two studies of children and adolescents with CP [49]. In a large RCT of children with unilateral CP (n = 102) participants demonstrated significant gains in ADL motor and processing skills, UL speed and dexterity, visual perception and self-perceived occupational performance after 20 weeks of Mitii™ training [49]. Also a pre-post study of 34 children with unilateral CP demonstrated significant gains in functional strength compared to aged matched controls after 20 weeks of training [50]. Both of these studies achieved significant improvement in primary outcomes across multiple domains even though the prescribed intervention dose was not achieved.

Virtual reality interventions, such as Mitii™ provide the opportunity for multi-modal training in home environments. A smaller, initial pilot study of investigating the efficacy Mitii™ in the home environment has demonstrated high adherence with over 85 % of participants achieving or exceeding the recommended total dose over 20 weeks of training (average dose of 74 h) [47]. In contrast, the dose achieved in the two larger studies in children with unilateral CP [49, 50] highlight the importance of considering participant engagement and motivational strategies during long home rehabilitation/treatment protocols. Strategies to ensure participant engagement during therapy are an important aspect to consider as they impact not only task performance but rehabilitation outcomes.

Extrinsic rewards may promote initial engagement in therapy whilst, intrinsic motivation is important for long-term maintenance and ultimately, change in attitudes and behaviour [51]. Extrinsic motivators are outside of the activity itself and may include rewards charts, non-tangible and tangible incentives [52, 53]. These rewards provide satisfaction and pleasure that the individual may not initially draw from the task. In contrast, intrinsic motivation involves the sense of satisfaction and pleasure from performing and completing activities [52]. These activities will be different for each individual and can include spending time with a friend whose company is enjoyed. Closely associated with intrinsic motivation is the act of setting goals and planning how to achieve these within a set timeframe [54]. In paediatrics this is often accomplished with parental involvement. Additionally, providing parents with strategies to maintain adherence also plays a significant role in a child’s motivation to perform and achieve goals [55].

Children with an ABI may have persistent physical, cognitive and psychosocial problems requiring outpatient therapy after discharge from hospital. The Mitii™ program potentially offers an intensive, home-based multi-modal training program which could increase the therapy dose achieved. The training program can be individualised and incremented according an individual’s level of ability – which is a distinct advantage over commercially available systems. Our proposed RCT of VR for children with an ABI proposes an adequately powered sample, using valid and reliable outcome measures (across all three domains of the ICF) with the assessment of retention effects post-intervention.

## Aims and hypotheses

The primary aim of this proposed study is to compare 20 weeks of Mitii™ training to usual care to improve ADL motor and processing skills, gross motor capacity, UL activity (unimanual and bimanual) and cognitive skills in a randomised, waitlist controlled trial for children and adolescents with an ABI. Secondarily, we aim to investigate the central neurovascular mechanisms underlying changes in UL function, motor planning and executive functions. This will be achieved by using functional magnetic resonance imaging (fMRI), structural imaging (sMRI), diffusion imaging (dMRI) and functional connectivity (FC) to measure central activation in the parts of the brain controlling movement. Understanding the foundation of neuroplasticity changes is essential to developing effective treatments with lasting effects. This proposed study aims to test the efficacy of Mitii™ across all three domains of the ICF (Table 1).

**Table 1:** Measures according to ICF domains

Impairment	Activity	Participation and Environment
*Classifier and Predictor Variables:*	*Primary Outcomes:*	*Classifier Variable:*
GMFCS, MACS, Edinburgh Handedness Inventory, Height, Range of Motion, Stereognosis, M2PD, Texture Tactile Perception, Mirror Movements, WISC-IV, Study questionnaire	AMPS	PEM-CY
	30 s rep_max_ of functional strength exercises	
*Secondary Outcomes:*	*Secondary Outcomes:*	*Secondary Outcomes:*
JTTHF	AHA	COPM
MUUL	6MWT	CASE
Executive Function: Score!, Sky Search and Sky Search DT from the TEA-Ch, Digit Span, Coding, and Symbol Search from WISC-IV; CTMT; TOL; Colour-Word Interference Test from D-KEFS; BRIEF; TVPS	HiMAT	CASP
	TUG test	Kidscreen-52
	4 day ActiGraph accelerometer	CHU-9D
		SDQ

Legend: AHA, Assisting Hand Assessment; AMPS, Assessment of Motor and Process Skills; BRIEF, Behaviour Rating Inventory of Executive Function; CASE, Child and Adolescent Scale of Environment; CASP, Child and Adolescent Scale of Participation; CHU-9D, Child Health Utility 9D; COPM, Canadian Occupational Performance Measure; CTMT, Comprehensive Trail-Making Test; D-KEFS, The Delis-Kaplan Executive Function System; GMFCS, Gross Motor Function Classification System; HiMAT, High-Level Mobility Assessment Tool; JTTHF, Jebsen Taylor Test of Hand Function; MACS, Manual Ability Classification System; MUUL, Melbourne Assessment of Unilateral Upper Limb Function; M2PD, Moving 2-Point Discrimination; PEM-CY, Participation and Environment Measure for Children and Youth; rep_max_, repetition maximum; SDQ, Strength and Difficulties Questionnaire; TEA-Ch, Test of Everyday Attention for Children; TOL, Tower of London test; TUG test, Timed Up and Go test; TVPS, Test of Visual Perception Skills; WISC-IV, Wechsler Intelligence Scale for Children – fourth edition; 6MWT, 6-min Walk Test

### Primary hypotheses

Compared to usual care, Mitii™ training will achieve significantly greater gains in:

1. Activities of daily living motor processing skills as indicated by an improvement of a minimum detectable change (MDC) of 0.5 logit scores on the Assessment of Motor and Process Skills (AMPS) immediately following training;
2. Gross motor capacity assessed by 30 s repetition maximum (rep_max_) of functional strength exercises (MDC = ≥28 repetitions on composite score of three functional strength exercises)

### Secondary hypotheses

Compared to usual care, Mitii™ training will achieve significantly greater:

1. Upper limb activity (unimanual and bimanual), as measured on the Jebsen-Taylor Test of Hand Function (JTTHF), MUUL and the Assisting Hand Assessment (AHA) [56];
2. Lower limb gross motor capacity assessed using the Timed Up and Go (TUG) test, 6-min Walk Test (6MWT) and the High-Level Mobility Assessment Tool (HiMAT);
3. Habitual physical activity (as measured on the ActiGraph® accelerometer) [57];
4. Neurovascular (functional MRI) changes;
5. Visual perception (visual discrimination, visual memory and visual sequential memory) measured on the Test of Visual Perception Skills (TVPS) [58];
6. Executive functioning including information processing, attentional control, cognitive flexibility, goal setting, working memory and behavioural manifestations in everyday life as measured by subtests from the Delis-Kaplan Executive Function System (D-KEFS), the Wechsler Intelligence Scale for Children Fourth Edition (WISC-IV), the Comprehensive Trail Making Test (CTMT), Tower of London – Second Edition and the Behaviour Rating Inventory of Executive Function (BRIEF);
7. Attention including performance on tasks of attention (TEA-Ch) and attention in everyday life as measured by parent rating (Conner’s 3);
8. Psychological functioning as measured by the Strengths and Difficulties Questionnaire (SDQ);
9. Performance and satisfaction with occupational performance goals using the COPM;
10. Participation as measured by the Child and Adolescent Scale of Environment (CASE) and Child and Adolescent Scale of Participation (CASP);
11. QOL on the domains of functioning and participation as measured on the Kidscreen-52;
12. Parent reported functional abilities in self-care and daily activities (using the MobQues28) [59]

Finally, Mitii™ will be more cost-effective compared with usual care as shown by resource use and consequence based on function (AMPS) and QOL (Child Health Utility 9D (CHU-9D) [60] and Kidscreen-52 [61])

## Methods/design

### Ethics

Full ethical approval for this study has been obtained by the Medical Ethics Committee of The University of Queensland (2013000212) and The Royal Children’s Hospital, Brisbane (HREC/12/QRCH/222). Written and informed consent will be obtained from all participants and their parents or guardians before entering the trial. This trial has been registered with the Australian New Zealand Clinical Trials Registry (ACTRN12613000403730).

### Recruitment

Sixty children at least 12 months post an ABI, which was acquired ≥28 days post full term birth will be recruited into the Mitii™ study. To be eligible, participants must be:

(i) Aged 8–16 years;
(ii) Independently ambulant at Gross Motor Classification System (GMFCS) equivalent level I or II [62];
(iii) Equivalent to a Manual Abilities Classification scale (MACs) of I to III [63];
(iv) Co-operative and have sufficient cognitive understanding, visual and verbal abilities to perform the assessment and training tasks;
(v) Classified with a brain injury which was acquired greater than 28 days after birth (based on the Australasian Rehabilitation Outcomes Centre impairment codes) [64]. For TBI, classified as mild complicated, moderate or severe brain injury;
(vi) Able to participate in the study over a period of 12 months;
(vii) Able to access the internet at home.

Children will be excluded if they have:

(i) A degenerative or metabolic condition;
(ii) Unstable epilepsy (i.e. frequent seizures not controlled by medication);
(iii) Undergone any surgical or any medical intervention impacting on UL and LL function in the past six months as this will confound results.

All children will undergo a comprehensive screen by a rehabilitation consultant to confirm eligibility. Participants will be recruited through referral by treating clinical staff (paediatricians, neuropsychologists physiotherapists, occupational therapists and speech therapists) from the Queensland Paediatric Rehabilitation Service (QPRS), Brisbane, Australia who will ask families if they are willing to be contacted regarding potential involvement in the study. Following verbal consent, study personnel will provide participant information and seek informed consent. To assist recruitment, participants will be reimbursed for travel expenses and flexible times and locations for assessments will be arranged.

### Design

**Fig. 1:**
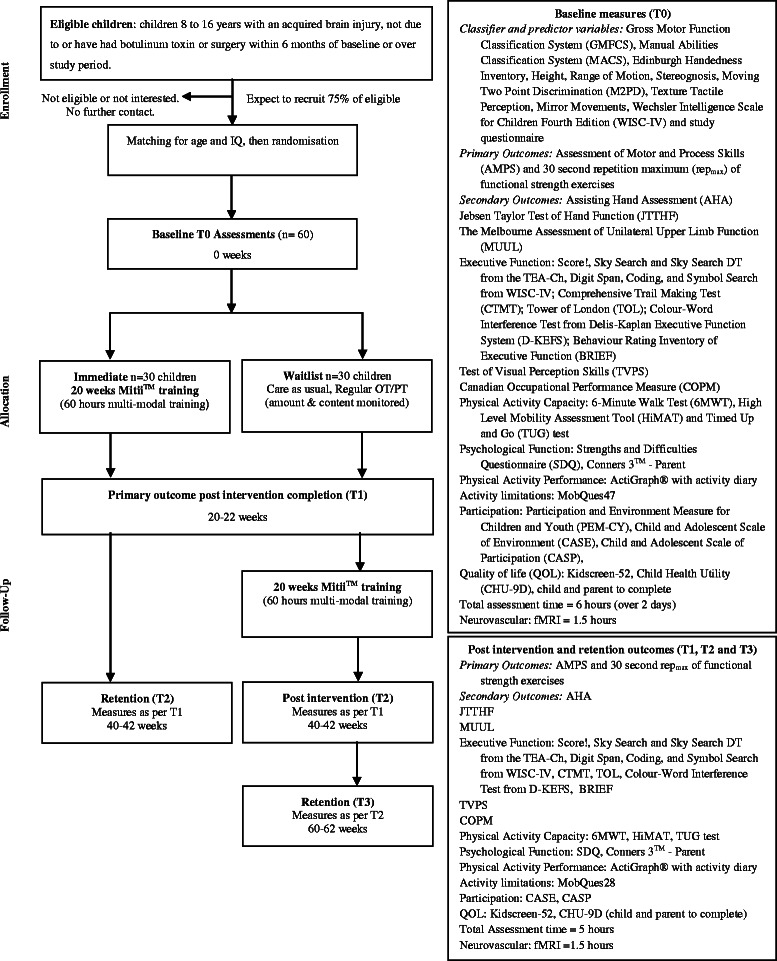
CONSORT Flow chart of the move it to improve it (Mitii™) study

This study utilises a matched-pairs, randomised waitlist controlled trial (see Fig. 1 for CONSORT flow chart). A matched pairs design was chosen to minimise the risk of baseline between-group differences. A waitlist design ensures that all participants have access to this novel intervention and will facilitate recruitment into the study. Within each pair, each participant will be randomised into either:

- *Immediate intervention group:* Families return home with Mitii™ equipment and commence training immediately for 20 weeks and then Mitii™ is withdrawn; or
- *Waitlist delayed intervention (control) group:* Families continue care as usual for 20 weeks, return to Brisbane for a one day re-assessment and then return home with Mitii™ equipment to commence training for 20 weeks. Care as usual includes any therapies (for example, occupational therapy and physiotherapy) usually provided by public or private services and visits to their treating paediatrician or general practitioner. Information about each participant’s usual care during the study period will be gathered using a general study questionnaire. After re-assessment the participants (and their family) will receive the Mitii™ introduction and equipment ready to commence training.

To limit confounding variables during the immediate intervention period (baseline to 20 week follow up), it will be requested that participants delay any of the following concomitant treatments – casting, splinting, intramuscular botulinum toxin type A [BoNT-A] injections (to UL or LL) and surgery. Participants will be able to continue other usual care therapies. Participants who have received intramuscular BoNT-A prior to beginning the study will have their baseline assessments postponed until one month after their standard follow up has been completed (approximately 10–12 weeks post injection). Questionnaires will record the type, frequency and duration of any concurrent therapies provided by public and private services during the study for all participants.

### Randomisation

Participants will be matched in pairs according to age (18 month age bands) and intelligence quotient (IQ) of ≤80 or >80 (measured on the Wechsler Intelligence Scale for Children – Fourth Edition) [65]. Where available an IQ assessment which has been completed greater than 12 months post injury will be used, otherwise an IQ assessment will be completed prior to matching. Each member of the pair will then be randomly allocated either number “1” or “2” using a computer generated number table. Treatment allocations will be recorded on a piece of folded paper inside a sealed, opaque envelopes and opened (in consecutive order) by independent, non-study personnel. Allocations will read either: “1: Waitlist 2: Immediate”, or “1: Immediate 2: Waitlist”. After the randomisation process is complete, study personnel will be informed of group allocation and information packs will be sent out to families.

### Blinding

Structural and functional MRI data will be qualitatively analysed by neurologists blinded to group allocation and order of assessments. Functional MRI training scan quality will be rated on region of activation, change over time and patterns of reorganisation. Data on the AHA and MUUL will be rated from video recordings masked and randomised.

### Adverse events

Any minor or major events associated with intervention or usual care groups will be screened at 20 weeks by open-ended questions. Any adverse events or unintended effects detected will be reviewed by the principal researchers RB, JZ and LM.

### Study procedure

To account for children’s varying abilities to attend and concentrate and, to mitigate fatigue the following scheduling strategies will be used to maximise data integrity during baseline and follow up assessments. Families will be contacted prior to baseline assessments to ascertain whether they feel their child will need one or two days to complete assessments. All participants will attend The University of Queensland, St Lucia campus for all assessments, minimising travel. Neuropsychological testing will be scheduled first as this requires most attention and concentration by participants. Regular breaks will be scheduled for participants between assessments as necessary at baseline and follow-up appointments. All primary outcome clinical assessments will be performed prior to Magnetic Resonance Imaging (MRI). Extra time will be allocated at the start and end of each day to manage any unforeseen complications which may arise (e.g. travel/time delays and fatigue issues). Participants in the immediate intervention group will spend an additional day for Mitii™ training and return home with Mitii™ equipment to begin the 20 weeks intervention. After baseline assessments (T0), the waitlist group will continue care as usual for 20 weeks. All participants will then return to The University of Queensland after 20 weeks for a one to two day re-assessment (T1). The waitlist group will spend an additional day for Mitii™ training before returning home with equipment to commence the intervention. For each participant data will be collected at Baseline (T0). The immediate intervention group will have follow up assessments conducted immediately post-training at 20 weeks (T1) and retention at 40 weeks post-baseline assessments (T2). The waitlist group will have a second baseline assessment at 20 weeks (T1), post-intervention at 40 weeks (T2) and retention of effects at 60 weeks post-baseline assessments (T3).

### Equipment

The Mitii™ intervention requires no specialist resources. All participants will receive a Microsoft Kinect®, laptops and pre-paid internet dongles for families who are unable to access a computer and/or internet at home for the 20 weeks intervention period. If families experience any technical issues, a study telemedicine engineer will be available to troubleshoot any technical difficulties. Step blocks, wobble discs and weights are also provided to families as an additional challenge for tasks as they progress through the program.

### Mitii™ intervention

Following occupational therapy, physiotherapy, neuropsychology and neurovascular assessments the participants and their parents will complete the Mitii™ training in preparation to start 20 weeks of intervention. Firstly, a therapist will deliver a lecture to explain the background and purpose of Mitii™, how the program works (see Additional file 1 for the participant manual) and motivational strategies to encourage engagement in the program (see Additional file 2 for child rewards chart). Children will then receive individualised training in how to use the Mitii™ program based on their capacity in baseline assessments. Therapists will select between an easy, moderate or difficult version addressing unilateral (right or left) or bimanual UL and LL deficits. A de-identified Mitii™ account will be created using an alias profile for each participant which will allow the program to be downloaded from the internet (https://mitiistorage.blob.core.windows.net/vsdeploy/clickonce/publish.htm) for each participant. Once the program has downloaded, the child will then able to log onto the program to complete their training at home or local environment at their convenience. The program will be requested to be completed six days a week for 20 weeks. This will provide a minimum potential treatment dose of 60 h. The participant should be appropriately supervised by a parent/guardian (according to their age) to ensure activities are being completed safely and correctly.

Once the participants and their parents have returned home, the three virtual trainers (one occupational therapist, one physiotherapist and one neuropsychologist) will develop an individually tailored program from the 12 available modules including; (1) gross motor or physical activity (e.g. repetitive sit-to-stand exercises); (2) combined cognitive and visual perception and; (3) UL (e.g. moving the UL to solve a mathematic equation) (See Additional file 3). Modules will be combined in sequence to make a daily program of approximately 30 min duration. To ensure each participant receives a similar training program, all daily programs will comprise of approximately 40 % gross-motor and 60 % cognitive-UL training modules.

The three virtual trainers will together evaluate each participant’s performance on a weekly basis and adjust module content variables to maintain the program at an appropriate level of difficulty or intensity to promote a training effect. Therapists access each participant’s program remotely via the Mitii™ user interface “Cockpit” and can monitor how frequently the participant has logged into Mitii™, how long they have spent engaged in the program, games that have been skipped and progress in each module. The complexity of the modules can be adjusted by increasing or decreasing the level of difficulty of visual perceptual (images graded on a colour code system of blue-green-red-black), cognitive (e.g. increasing the length of a memory sequence) and motor activities (e.g. reducing the base of support for balance).

### Motivational strategies

Motivational strategies will be discussed during the initial Mitii™ training session with families and will include weekly performance feedback, positive re-enforcement by parents/guardians and incorporating Mitii™ into the regular family routine. Each participant will also have the option of using a rewards chart (child or adolescent version) broken up into four, five week blocks throughout the program as an additional motivational strategy (see Additional file 2 for child rewards chart). The rewards chart allows small rewards to be negotiated with the participants and their parents and/or guardians at the completion of each stage during the initial Mitii™ training. Program options such as physiotherapists demonstrating aerobic activities that are used during activity modules and including real-world images in figure ground, spatial relations, match two images and memory modules, are more likely to appeal to teenagers will be incorporated in their programs. One of the three virtual trainers will be assigned as the main contact for the participant and their family and remain in weekly contact. Families will be contacted weekly (or at the family’s request) to encourage ongoing engagement in the program. Once participants have completed the 20 weeks intervention they will receive a small reward provided by study personnel and parents will be asked to complete an exit questionnaire.

### Data management

Data will be recorded and managed through a confidential online database run through the Queensland Cerebral Palsy and Rehabilitation Research Centre (QCPRRC). Participants will also have paper files which will be de-identified and only contain the participant’s unique identification code. These files will be stored in a locked filing cabinet at the QCPRRC separate from files containing participant demographic and consent information. The Mitii™ program, participants’ Mitii™ scores and training history will be stored on a secure server at the Microsoft Azure Data Centre in Ireland. In addition to Microsoft’s standard data procedures, the Mitii™ program and associated data will be backed up every two weeks. To ensure participants’ confidentiality, participants will not have personal information or details recorded on the program. Participants will use a non-identifying “alias” name to log into Mitii™ and study personnel will use their personal email (not linked to the Mitii™ program) for correspondence.

## Classification measures

### Australasian Rehabilitation Outcome Centre (AROC) impairment codes

Classification of the ABI will be made according to the AROC impairment codes [64]. The AROC impairment codes describe the reason for rehabilitation according to impairment groups. Episodes are classified according to the primary reason for the current episode of rehabilitation care. In total, there are sixteen impairment groups. Two groups relate to rehabilitation episodes for children with ABI – Stroke and Brain Dysfunction. Firstly, the Stroke impairment group can be subdivided into two sub-groups, “Haemorrhagic” and “Ischaemic”. These sub-groups are then further subdivided into individual group codes. Secondly, Brain Dysfunction can be divided into two sub-groups including, “Non-traumatic” and “Traumatic”. Non-traumatic brain dysfunction codes include subarachnoid haemorrhage (2.11), anoxic brain damage (2.12) and other non-traumatic brain dysfunction (2.13). Examples of other non-traumatic brain dysfunction include brain injury as a result of tumour, encephalitis or complications of ventriculoperitoneal shunt revisions. Traumatic brain dysfunction codes can be open (2.21) or closed injury (2.22). A rehabilitation consultant from QPRS will classify all participants according to these impairment codes.

### Classification of the brain lesion

The nature of the brain lesion will be classified on sMRI using both a qualitative description [66] and semi-quantitative scale for classification of brain lesion severity for children with ABI [67, 68].

Structural MRI will be obtained for each child using a 3 Tesla (3T) Magnetom (Siemens) scanner using T1-weighted high resolution 3D MPRAGE, T2-weighted FSE and 3D FLAIR sequences. Structural MRI will be evaluated by a paediatric neurologist (SF who will be blinded to clinical and medical history) to describe the type of lesion and presumed pathogenic pattern (e.g. stroke, hypoxia/anoxia, toxic, metabolic or infective injuries and head trauma).

The brain lesion severity will be determined by using a semi-quantitative scale designed for classification of brain lesion severity in children with CP [67]. The semi-quantitative scale has demonstrated high inter-rater and intra-rater reliability [67]. According to the scoring system, the brain lesion is represented on a graphical template and raw scores for lobes, subcortical structures (basal ganglia, thalami and brainstem), corpus callosum and cerebellum are systematically calculated, where higher scores represent more severe pathology. Each hemispheric score (HS) is the sum of the lobar scores (maximum score of 12). The basal ganglia and brainstem score (BGBS) is the sum of left and right subcortical structures (basal ganglia, thalamus, brainstem and posterior limb of the internal capsule) (maximum score of 10). The global score (GS) is the sum of the right and left HS, BGBS, corpus callosum and cerebellum scores (maximum of 40). A laterality index (LI) will be calculated for each scan (range 0–1) to determine the lateralisation of the lesion, assuming the HS as the lateralised measure. A score approximating 0 indicates a more bilateral lesion; a score approaching 1 more unilateral involvement. Preliminary to further analysis, reliability will be tested in children with an ABI.

### Classification of brain injury severity

Where possible, available information will be collected from the participant’s medical records regarding their initial brain injury as these may have a prognostic effect. Information will include Glasgow Coma Scale (GCS) at the time of injury or admission; in the instance of a TBI length of Post Traumatic Amnesia (PTA), loss of consciousness (LOC) and duration of coma; treating hospital and length of hospitalisation; additional injuries or complications at time of injury and the Functional Independence Measure for Children (WeeFIM) at the time of discharge [69–71].

A description of comorbidities (e.g. orthopaedic injury, epilepsy, psychological and pre injury status) and any other treatments and interventions (e.g. surgical, medical or radiation) will be collected on an entry questionnaire completed by the parent. Information regarding the participant’s school grade and involvement in a special education program will also be collected in the same baseline questionnaire.

### Gross Motor Function Classification System (GMFCS)

The GMFCS for children with CP classifies how children perform self-initiated movements based on sitting (truncal control) and walking across a five levels [72]. Construct validity has been obtained with a correlation between the GMFCS and the Gross Motor Function Measure (GMFM) of *r =* 0.91 [72]. The measure also has good inter observer reliability between professionals and professionals and parents in children with CP [73]. This classification measure has not been validated for use with children who have an ABI however it is expected that all children in the sample will be equivalent to a GMFCS I (walks without limitations) or II (walks with limitations). Children will be classified by a physiotherapist.

### Manual Abilities Classification System (MACS)

The MACS is a five level classification of how well children with CP use their hands to handle objects in day-to-day activities [63]. The MACS has reported construct validity and excellent inter-rater reliability (Intra-class correlation coefficients [ICC] = 0.97 between therapists and ICC = 0.96 between therapists and parents) for children with CP [74]. This scale has not been validated for children with an ABI however, it is expected that all children included in this study will be equivalent to MACS level I (able to handle objects easily and successfully), level II (able to handle most objects but with somewhat reduced quality and/or speed of achievement so that alternate ways of performance might be used) or level III (handles objects with difficulty; needs help to prepare and/or modify activities). Children will be classified by an occupational therapist.

### Edinburgh Handedness Inventory

During baseline assessments, hand dominance will be assessed using the The Edinburgh Handedness Inventory [75]. The Edinburgh Handedness Inventory questionnaire consists of 10 items regarding hand preference (right or left) in performing a number of everyday tasks requiring one (writing, drawing, throwing and using scissors) or two (e.g. using a broom or opening a box) hands. A laterality quotient is calculated using the following formula: laterality quotient = (right hand – left hand/(right hand + left hand)*100).

### Passive Range of Motion (ROM)

Passive ROM for the unimpaired and impaired side will be assessed by the same occupational therapist (UL) and physiotherapists (LL) at baseline only to determine any relevant impairments (e.g. joint contractures) which may limit access to the program and inform participation in the study.

### Sensory measures

Three different descriptive, sensory measures will be obtained from participants at baseline by an occupational therapist:

I. Stereognosis will be assessed on the non-dominant and dominant hands using the approach described by Feys [76]. Three familiar objects (teaspoon, key and peg) and six similar matched objects (safety pin and paperclip; pen and pencil; coin and button) will be used. With vision occluded, participants will be presented with each item. If a participant is unable to grasp, manipulate or release an object the occupational therapist will assist the participant and move the object for them within their hand. A corresponding set of items will be used to allow participants to identify the object in order to minimise any errors due to incorrect naming of the object. Scores range on a scale from 0–9, where participants scoring below 9, will be considered to have impaired stereognosis [77].
II. Moving two point discrimination (M2PD) will be measured using the Disk-criminator® (Baltimore, Maryland) on both the non-dominant and dominant hands. Either one or two points will be randomly applied in continuous moving firm contact longitudinally to the pulp of the index finger with vision occluded [78]. The minimum distance participants can usually distinguish between two discrete points, ranges from 2mm (normal) to 15 mm (poor) [79].
III. Texture Tactile Perception will be tested using the AsTex Perspex board that displays tactile gratings of reducing tactile discrimination index [80]. Starting at the “rough” end of the board, movement of the participant’s index finger, then thumb, then fifth finger will be guided by the examiner along the board at a constant speed in a standardised manner. Participants will be instructed to stop immediately when the board feels smooth (gratings become too close together to determine their separation). Each point will be recorded, with the final outcome the average of three trials for each digit. The average scores will be converted to the tactile discrimination index for each finger using the chart available with the test kit.

### Mirror movements

The presence of mirror movements will be assessed (by an occupational therapist) and scored on the side of the body unintentionally performing the movement during three unimanual UL tasks: (i) rapid tapping of the index finger on the distal thumb, (ii) alternating supination and pronation of the forearm and (iii) repetitive alternate touching of each fingertip to the tip of the thumb of the same hand, in order. Participants will be scored on a four point scale ranging from no clearly imitative movements, to movement equal to that of the intended hand. Possible total score range is from 0–12 [81].

### Grip strength

Grip strength will be measured using a hand held dynamometer (Smedley®, Scientific Instruments Co Ltd) by an occupational therapist. Grip strength will be measured for three attempts on each UL (kilograms force, Kgf) according to the guidelines of the American Society of Hand Therapists [82]. The mean of the three attempts will be used to compare limbs and to evaluate changes over time.

### Anthropometric data

Height will be measured to the nearest 0.5 cm while the participant is standing with his or her back against a wall with a ruler. Body Mass Index (BMI) will be collected at baseline only. This will be used to convert activity capacity tests into referenced percentiles.

### Wechsler Intelligence Scale for Children–Fourth Edition (WISC-IV)

Overall level of cognitive intellectual functioning will be assessed using the WISC-IV [65]. The WISC-IV consists of ten subtests that make up the four indices of verbal comprehension (VCI), perceptual reasoning (PRI), working memory (WM), and processing speed (PSI). A full scale intelligence quotient (FSIQ) score can be calculated from these index scores. The VCI index is made up of the Vocabulary, Comprehension and Similarities subtests. The Vocabulary subtest assesses knowledge of word meaning and will require participants to name pictures or provide spoken definitions of words (e.g., “what is a bicycle?”). The Comprehension subtest measures verbal reasoning and conceptualisation and demonstrates practical information. The Similarities subtest assesses verbal abstraction and reasoning and will require participants to describe how two words are similar (e.g., “how are anger and joy alike”). The subtests of Block Design, Picture Concepts and Matrix Reasoning make up the PRI index. Block design will require participants to construct abstract visual designs using a set of red-and-white three dimensional blocks within a specified time period. Picture Concepts is designed to measure nonverbal abstract, categorical reasoning ability. For Matrix Reasoning, participants will be shown an array of visual designs with one missing square. They will be required to use their non-verbal abstract reasoning and problem solving skills in order to select the correct picture from an array of five options that fits into the missing space of a visual design. The WMI index is made up of the Digit Span and Letter-number sequencing subtest. For the Digit Span subtest, participants will be required to repeat a number of verbally presented digits in both the forward and reverse order. The Letter-number sequencing subtest will require participants to sequence letters and numbers in order which involves sequencing, mental manipulation, attention and short term auditory memory. Finally, the PRI index is made up of the Coding and Symbol Search subtests. For the Coding subtest, participants will need to match up abstract symbols with numbers from a key and for Symbol Search, participants will be required to detect the presence of one or more symbols in a sequence of five. These subtests have a two-minute time limit and therefore require participants to work as quickly as possible without making mistakes [65].

Raw scores from the subtests can be converted into scaled scores with a mean of 10 and standard deviation (SD) of three using age and gender appropriate normative data. Index scores can be converted into composite or scaled scores with a mean of 100 and SD of 15. The VCI, PRI and WMI and FSIQ index scores of the WISC-IV demonstrate very high levels of internal consistency (α > 0.90). The PSI index has high internal consistency (α = 0.80-0.89) [65].

## Neurovascular measures

### Whole-brain fMRI studies

All participants will be checked for 3T MRI safety including no metal implants or ventriculoperitoneal shunts. Participants will be prepared in the mock MRI scanner prior to the real scan to familiarise them with the image procedure and minimise the potential for psychological distress.

Functional imaging at 3T on a Siemens MAGNETOM Trio MRI scanner will be conducted on the research dedicated scanner at The University of Queensland, Centre for Advanced Imaging (CAI), Brisbane, Australia. Published methods will be utilised for conducting serial functional MRI (fMRI) studies including preparation in a mock MRI scanner and assessing activation during a range of tasks as outlined below. Two of three tasks will be performed, depending on age and motor abilities:

(i) All participants will perform a simple wrist flexion/extension movement task in a two-condition block design, visually cued via instructions projected on a screen. The baseline condition is no movement (rest). A recording of a metronome at two hertz will provide an auditory cue for the rate of movement. Participants will be required to flex and extend the wrists of each hand one at a time, in time with metronome and lie still with no movement during the baseline blocks. The task and rest periods are 30 s with the activation cycle repeated four times. The movements performed in the scanner will be rated for speed, ROM, ability to isolate the movement and presence of mirror movements in the contralateral hand. If there is task-correlated breath-holding, the task will be repeated.
(ii) Older participants and those with sufficient fine control in the least impaired hand will also perform a complex movement sequencing task, aimed at examining function in higher motor areas and basal ganglia circuits. In this task, visual cues will indicate a sequence of three button-presses to perform (e.g. 132; sequence condition), or three simple repetitive presses with the index finger only (e.g. 111; simple condition). The task will be run in blocked design, with 20 s of simple, 20 s of sequence, and 20 s rest blocks in counterbalanced order for a total scan duration of six minutes.
(iii) For examination of visuo-spatial processing and spatial working memory, a mental rotation task will be used [83]. For each trial, participants will be presented with a single target stimulus above fixation and two-test stimuli below, on the visual display. Participants will be required to indicate by button-press which test stimulus matches the target. Mental rotation stimuli are Shepard–Metzler three-dimensional cube objects, with target and test stimuli differing by 30° or 60° rotation (two dimensional rotation only), as used previously in children of the same age range [83]. In a baseline condition, participants must indicate which of two spatial Fourier-transformed ‘noise’ patches is the best visual match to the target. Stimuli are presented for 10 s with one second inter-stimulus interval. Groups of three baseline trials alternating with three rotation trials (forming 33 s blocks) are presented in 12 blocks over a total scan duration of six minutes and 36 s. The mental rotation task is similar to matching puzzle pieces, which some children may find interesting. The mental rotation task has been performed in paediatric populations with Attention Deficit Hyperactivity Disorder (ADHD) [83]. As the mental rotation task is quite different to the current battery of occupational therapy and neuropsychological tests, children will perform the task both inside and outside the scanner to compare performance and account for practice effects. An additional five minutes of resting-state fMRI will also be collected for analysis of FC. Tasks performed prior to resting-state fMRI can influence FC so the resting-state data will be collected after the motor paradigms [84].

For all tasks, fMRI will be acquired using a BOLD acquisition sequence (Gradient-recalled-echo [GRE] Echo-Planar Imaging [EPI], Repetition Time [RT] = 3.0 s, Echo Time [ET] = 30 milliseconds, flip angle = 850, slice thickness = 3 mm, Field of View [FOV] =216 mm, 44 slices, 72 × 72 matrix yielding an in-plane resolution of 3.0 mm × 3.0 mm). A single set of T2-weighted anatomical, FLAIR and 3D T1 (MPRAGE 1mm isotropic resolution) volumes will also be collected. Functional MRI image processing, analysis and visualisation will be performed using SPM software (Welcome Department of Imaging Neuroscience, London, UK).

### Diffusion imaging acquisition and white matter fibre tracking

Diffusion-weighted images will be acquired using a twice-refocused single-shot EPI sequence (64 directions, B-value = 3000 s per millimetre squared (s/mm^2^), contiguous slices with whole-brain coverage at 2.2 mm isotropic resolution, acquisition time approximately 10 min). White-matter tractography will be undertaken in a manner robust to crossing fibres, using constrained spherical deconvolution (CSD) and probabilistic streamlines using MRtrix software [85–87].

To improve the understanding of cortical plasticity post-intervention, cortical reorganisation will be investigated using a combined fMRI-probabilistic tractography approach. White matter connectivity maps will be produced using MRtrix by seeding every brain voxel and using regions of corticomotor activation generated post-intervention as target masks for the tracking algorithm. This will enable identification of all cortical regions, which have white matter tracts that directly project into corticomotor regions exhibiting plasticity as a result of the motor training paradigms. By comparing connectivity maps pre and post-intervention (registered within the same image space), this strategy enables both an anatomical view of cortical reorganisation and quantitatively analyses altered connectivity by measuring the number of streamlines connecting two cortical regions, corrected for brain size. The basis of this strategy has recently been published in Neuroimage [88]. It is hypothesised that increased intrahemispheric and interhemispheric connectivity showing recruitment of additional corticomotor areas will directly correlate with improved motor function [88]. The HARDI paradigm for this cohort of children is the same as previously conducted at CAI in the Mitii™ CP and CoMBIT protocol studies [89, 90].

Nine typically developing children will also be recruited as a reference sample for the neuroimaging studies. Children will be recruited through staff newsletters and from other studies within the centre. Five children will complete all tasks involved for fMRI, sMRI and dMRI. Four children will only complete sMRI and mental rotation tasks.

## Primary outcome measures

### Assessment of Motor and Process Skills (AMPs)

The AMPs assesses the quality of performance of an individual engaged in culturally relevant personal or instrumental ADL. It is a standardised, criterion-referenced, observational assessment that can be used for children from two years of age [91]. An interview is conducted with the child and the therapist selects appropriate familiar tasks from 116 task options. The participant performs a minimum of two daily activities (e.g. dressing, eating, and food preparation). A trained occupational therapist will evaluate the participant’s performance on degree of exertion, efficacy, confidence and independence for 16 ADL motor and 20 ADL processing skills, as well as overall functioning levels. Performance in each of the motor and processing skills will be scored from 1–4 (1 = deficient performance that *impeded* the action progression and yielded unacceptable outcomes, to 4 = competent performance that *supported* the action progression and yielded good outcomes). Computer-scoring software will be used to convert raw scores into linear ADL motor and ADL process ability measures through many-faceted Rasch analyses. Scores range from +4 to −3 for motor skills and +3 to −4 for processing skills. Test-retest reliability of the AMPS is high for both motor (ICC = 0.93; 95 % CI = 0.86-0.97) and process (ICC = 0.86, 95 % CI = 0.65-0.94) skill scales in children with CP [92]. The minimum detectable change was found to be 0.23 logits for the AMPS motor scale and 0.30 for the AMPS process scale [92]. This measure is also very sensitive to change, as it evaluates the smallest possible units of ADL task performance and involves 116 task options which vary in challenge. Participants will have the option to participate in test-retest reproducibility, carrying out the AMPS over two consecutive assessment days with a trained occupational therapist.

### Lower Limb (LL) functional strength

Thirty second repetition maximum (rep_max_) of functional strength exercises (including sit-to-stand, lateral step-ups and half-kneel to stand) will be tested according to published recommendations [93]:

Sit-to-stand: This test measures the ability of the participant to attain standing (from 90° knee flexion) as many times as possible in 30 s with arms free or placed on their hips and without support from the chair or their body. Height of the chair will be defined by the ability of the participant to attain 90° hip and knee flexion with feet flat on the floor. One repetition will be defined as each time the participant is within 15° of hip and knee extension, visually estimated by a physiotherapist.

Lateral Step-Up: The aim of this test is to perform as many step up repetitions onto a 20 cm step block as possible in 30 s. Participants will be asked to stand with the foot being tested on the step and the non-test leg on the floor, parallel and shoulder width apart. The participant then raises the non-test foot off the ground until the test leg has reached within 10° of knee extension (visually estimated) for the tested foot during the extension phase of the test. The non-test foot does not have to touch the step block. Repetitions will be counted each time the heel or toes of the foot not being tested touches the ground. Both legs will be tested with the dominant leg tested first.

Half-kneel to Stand: This activity tests the ability of participants to attain a stride stance through a half-kneel position without the use of arms for as many repetitions that can be completed in 30 s. The participant is placed in half-kneel with buttocks clear of the lower legs and floor. The participant will be instructed to assume a standing position within 15° of knee extension (visually estimated) and remain with their feet apart (one repetition). This is a slight adaptation to the original protocol where participants assumed a standing position with feet together. The dominant leg in front will be tested first and repeated for the non-dominant side.

For each LL functional strength exercise participants will be given verbal and visual instructions as well as two practice repetitions prior to testing. The exercises will be assessed in the following order: (i) Sit-to-stand, (ii) Lateral step-up dominant, (iii) Lateral step-up non-dominant, (iv) Half-kneel to stand dominant and (v) Half-kneel to stand non-dominant and participants will be given verbal encouragement throughout. Participants will be given 180 s rests between exercises. If a participant cannot complete an exercise whilst performing the practice attempts, they will be assigned a score of 0 and will not proceed to testing.

Functional strength tests demonstrate acceptable inter-tester reliability (ICC= > 0.91; coefficient of variation (CV) 12.1-22.7 %) in 25 children with CP [93]. Reliability for each of these three tests was also strong (Lateral step up ICC = 0.94; Sit to stand ICC = 0.91; Half kneel to stand ICC = 0.93-0.96) [93]. Mean repetitions for each of the functional measures were as follows: (i) sit-to-stand was 14.4 (SD = 5.0; Standard error of measurement [SEM] = 1.5 reps; CV = 12.1 %), (ii) lateral step up left were 13.2 (SD = 10.5; SEM = 2.4 reps; CV = 17.8 %), (iii) Lateral step-up right was 12.6 (SD = 10.4; SEM = 2.6 reps; CV = 22.7 %), (iv) Half-kneel to stand left was 7.5 (SD = 5.5; SEM = 1.1 reps; CV = 28.6 %), (v) Half-kneel to stand right was 6.0 (SD = 5.3; SEM = 1.4 reps; CV = 39.9 %) [93].

## Secondary outcome measures

### Body structure and function domain

#### Jebsen Taylor Test of Hand Function (JTTHF)

Upper limb manual speed and dexterity will be measured using the JTTHF [94]. Six timed tasks of varying complexity using everyday items requiring grasp and release ability will be performed on each UL. The dominant hand will be tested first, followed by the non-dominant hand. In accordance with previously published modifications to the administration of the JTTHF, the writing task will be omitted. The maximum allowable time to complete each item will be capped at two minutes to reduce frustration [95–97]. The JTTHF is responsive to change due to UL intervention although there have been questions raised about the stability of test-retest performance in the unimpaired limb [95–98]. Inter-rater reliability for each subtest is high (ICC = 0.82-1.0).

#### The Melbourne Assessment of Unilateral Upper Limb Function (MUUL)

Quality of UL movement will be evaluated using the MUUL [99]. The MUUL was designed for children with neurological impairment aged 5–15 years and comprises 16 criterion-referenced items measuring movement range, accuracy, dexterity and fluency. The maximum possible raw score is 122. Raw scores will be converted and reported as percentage scores (higher scores represent greater quality of movement). Total test scores have very high levels of inter-rater (ICC = 0.95), intra-rater (ICC = 0.97), and test re-test reliability (Concordance Correlation of 0.98). Construct, content and criterion validity for the MUUL have been established [99–101]. The MDC has been estimated in a number of studies ranging from 7.4 to 14 % [99, 102, 103]. The MUUL has recently undergone revision. Rasch analysis did not support uni-dimensionality of the MUUL. The revised version comprised 14 items that are organised in four separate sub-scales [104]. The original version of the MUUL will be used in this study as evidence for the revised version is preliminary.

#### Executive functioning

Executive functioning will be evaluated using a comprehensive neuropsychological test battery which was designed to assess the four domains of executive functioning in accordance with Anderson’s paediatric model of executive functioning [13]. The four domains are attentional control, information processing, cognitive flexibility and attentional control. The neuropsychological test battery consists of subtests from the Delis-Kaplan Executive Function System (D-KEFS) [105], Test of Everyday Attention For Children [106], the Wechsler Intelligence Scale for Children Fourth Edition (WISC-IV) [65], the Comprehensive Trail Making Test (CTMT) [107] and the Tower of London – Second Edition [108]. The Behaviour Rating Inventory of Executive Function (BRIEF) [109], a parent-rated questionnaire will be used as a measure of behavioural manifestations of executive functioning in everyday life. All scores will be converted into scaled scores using age and gender based norms.

i) *Colour-Word Interference Test (from the D-KEFS)*

The inhibition condition of the Colour-Word Interference subtest of the D-KEFS will be used as a primary measure of attentional control. The subtest measures an individual’s ability to suppress an automatic response in favour for an abstract one. Participants will be assessed on their ability to name the colour of the ink colour words that are printed across five rows (e.g. say “blue” for the word “red” that is printed in blue ink). Participants will be timed (in seconds) on how long it takes them to complete the task as well as how many mistakes they make. Raw scores are converted into scaled scores (M = 10, SD = 3) using normative data provided in the manual. The Colour-Word Interference subtest of the D-KEFS has demonstrated excellent test-retest reliability (*r* = 0.90) [105].

ii) *Comprehensive Trail Making Test (CTMT)*

Trails one, two and three from the Comprehensive Trail Making test will be used to measure attentional control and trails four and five will be used to measure cognitive flexibility in children with an ABI. In trails one to three, participants will connect numbers printed on an A4 sheet in numerical order from 1–25. Distractor items are contained in trails two and three to increase the need for attentional control. In trail four, participants will draw a line in order connecting the numbers 1–20. The numbers are presented in both Arabic numerals (e.g. 1, 7) and numbers spelled out in the English language form (e.g. Nine). Lastly, for trail five, participants will be required to switch back and forth between connecting numbers in numerical order and letters in alphabetical order, also printed on an A4 sheet, from 1–13, and A – L (e.g. “1-a-2-b-3-c”). The total time (seconds) taken to complete each trail will be recorded, with a longer time indicating greater difficulty with attentional control or cognitive flexibility. Raw scores will be converted to T-scores (M = 50, SD = 10). Good internal test-retest reliability has been shown for the five trails (*r* = 0.70–0.78) [107].

iii) *Tower of London (TOL) – Second Edition*

The Tower of London will be used to measure goal setting. Participants will move three coloured beads across three vertical pegs on their board varying in height to build a target tower on the examiner’s identical board, within a specified time limit. There are 10 problems of increasing difficulty. They will be instructed to use the least number of moves possible to complete the tower, that they can only move one bead at a time and that they must not put more beads on a peg than it will hold (e.g. the second peg can only hold two beads and the third peg can only hold one bead). The total move score, which is based on the total number of moves needed to build each of the 10 towers; the total correct score which is the number of towers correctly solved in the recommended moves; and the total rule violations will be used to measure goal setting abilities. The lower the total move score and total correct score, and the higher the total rule violations score indicate greater goal setting difficulties. Raw scores will be converted into standard scores (M = 100, SD = 15). The Tower of London has adequate test-retest reliability (*r* = 0.28–0.75) [108].

iv) *Digit Span (from the WISC-IV)*

As explained previously, the Digit Span subtest from the WISC-IV is a verbal memory task where children are required to repeat a number of digits in the forward and backwards order. Digit Span Forward is a measure of a child’s ability to temporarily store information. The examiner will say a string of numbers increasing from two digits to nine, and the participant is required to repeat them back. Digit Span Backwards, is similar but instead of repeating the string of numbers in the same order as presented, the child is required to repeat the string of numbers in the reverse order (e.g. if “5-7-4”, the child should say “4-7-5”). Digit Span Backward is also a measure of a child’s ability to temporarily store information but they are also required to mentally manipulate in working memory too. As such, Digit Span Backwards will be used as a measure of cognitive flexibility. Children are given a score of one for every number string they repeat correctly in reverse order. This means lower overall scores for Digit Span Backwards will indicate poorer cognitive flexibility. Raw scores are converted into scaled scores (M = 10, SD = 3) using normative data provided in the manual. Internal consistency for Digit Span Backwards was good (α = 0.80) and it also has adequate test-retest reliability (*r* = 0.74) [110].

v) *Coding (from the WISC-IV)*

The Coding subtest from the WISC-IV will be used as a measure of information processing. Participants will be required to match up and copy abstract geometric shapes with numbers from a key within a two minute period. Participants will be scored based on the number of correctly copied abstract geometric shapes within the time limit. Lower numbers will ultimately indicate poor information processing abilities. Raw scores will be converted into scaled scores (M = 10, SD = 3) using normative data provided in the manual. Coding has been shown to have a good internal consistency (α = 0.82) and test-retest reliability (*r* = 0.81) [110].

vi) *Symbol Search (from the WISC-IV)*

The Symbol Search subtest of the WISC-IV is also used as a measure of information processing. In a two-minute time limit, participants will be required to visually scan for target symbols in groups of five symbols and indicate whether the target symbol is in the group by placing a line through the word ‘yes’ or ‘no’. Participants will be scored based on the total number of correctly identified symbols minus the total number of incorrectly identified symbols. Lower scores on Symbol Search indicate poorer information processing. Raw scores will be converted into scaled scores (M = 10, SD = 3) using normative data provided in the manual. Symbol search has adequate internal consistency (α = 0.79) and a high level of test-retest reliability (*r* = 0.80) [110].

vii) *Brief Rating Inventory of Executive Function (BRIEF)*

As indicated, the BRIEF is a parent-rated questionnaire designed to assess the behavioural manifestations of executive functions in everyday life. Parents will be required to rate 85 items (e.g. “becomes upset with new situations”) on a three-point likert scale ranging from 1 (*never)* to 3 *(often)*. Raw scores will be converted into *T* scores (M = 50, SD = 10) using normative data provided in the manual. The BRIEF consists of eight subscales which combine to form the Behavioural Regulation Index (BRI; Initiate, Working Memory, Plan/Organise, Organisation of Materials and Monitor subscales) and the Metacognition Index (MCI; Inhibit, Emotional Control, and Shift subscales). These inturn form the overall Global Executive Composite (GEC). Elevations on subscales and indices will be determined by a *T* score of 65 and above, which is 1.5 SD above the mean. Higher *T* scores indicate progressively greater levels of executive dysfunction. The BRI, MCI and GEC indices will be used as primary measures of executive function in everyday life. The BRIEF has been shown to be a valid measure of executive functioning and has good internal consistency (*α* = 0.80-0.98) and high test-retest reliability on the BRI (*r* = 0.92), MCI (*r* = 0.88), and the GEC (*r* = 0.86) [109].

#### Attention

Attention will be measured by using the Test of Everyday Attention for Children (TEA-Ch) [106] and the parent rated questionnaire the Conner’s 3^rd^ Edition. Selected subtests from the TEA-Ch will be used and are described below.

#### Test of Everyday Attention for Children [106]

viii) *Score! (TEA-Ch)*

Sustained attention will be assessed using Score!. Participants will be required to keep a count of the number of ‘scoring’ sounds they hear on a tape, as if they were keeping the score on a computer game across several trials. Due to the ceiling effect in this test, correlations are unrealistic therefore there is 76.2 % agreement within one SD for test-retest [106].

ix) *Sky Search (TEA-Ch)*

Selective/focused attention will be assessed using Sky Search. This is a brief, timed subtest. Participants will be required to circle as many ‘target’ spaceships as possible on an A3 sheet filled with very similar distracter spaceships. In the second part of the task there are no distracters. Subtracting part two from part one gives a measure of a child’s ability to make this selection that is relatively free from the influence of motor slowness. Sky Search has high test-retest reliability (*r* = 0.80 for time-per-target and *r* = 0.75 for attention score) [106].

x) *Sky Search DT (TEA-Ch)*

Sustained attention will be assessed using Sky Search Dual Task (DT). This subtest combines the sustained attention task of counting ‘scoring’ sounds (Score!) with the selective/focused attention task of Sky Search. Participants will be required to circle as many ‘target’ spaceships as possible on the A3 sheet at the same time as counting the number of ‘scoring’ sounds on a tape. Sky Search DT has high test-retest reliability (*r* = 0.81) [106].

#### Conners 3^rd^ Edition™ (Conners 3™)

The Conners 3™ [111] is a thorough assessment of ADHD and its most common co-morbid problems and disorders in children and adolescents ages 6–18 years old. The Conners 3™ will be completed by the participant’s parents or guardian and consists of 110 statements and takes approximately 20 min to complete. Parents or guardians must rate each statement using a four-point scale ranging from ‘0 – Not true at all (never, seldom)’ to ‘3 – Very much true (very often, very frequently)’. The Conners 3™ measures the seven key areas of inattention, learning problems, aggression, family relations, hyperactivity/impulsivity, executive functioning and peer relations. Raw scores are converted into T scores based on a large representative normative sample based on United States of America consensus data. In addition, the Conners 3™ calculates T scores for symptom scales including ADHD Hyperactive/Impulsive, ADHD Combined, Oppositional Defiant Disorder, ADHD Inattentive and Conduct Disorder. Both internal consistency coefficients (α = 0.83–0.94) and test-retest reliability (*r* = 0.52–0.94) are good for the Conners 3™ Parent version total sample age range [111].

#### Test of Visual Perceptual Skills (TVPS)

The TVPS assesses visual-perceptual strengths and weaknesses on seven subtests (visuo-spatial relationships, visual discrimination, visual memory, visual sequencing memory, visual closure, visual constancy and visual figure ground) each with developmentally incremented items. Performance will be determined by number of correct answers in each test (maximum of 16 in each of the seven tests). Raw scores will be scaled and converted into percentage score for the age group based on normative data [58]. A systematic review of visual perception tools of children with hemiplegia, found strong test-retest reliability for total TVPS scores [112].

### Activity capacity domain

#### 6-Minute Walk Test (6MWT)

The 6MWT is an objective, submaximal evaluation of functional exercise capacity. It reflects an exercise level close to that of daily life activities. This test measures the distance that a patient can quickly walk on a flat, hard surfaces in a six minute period (the 6MWD) [113]. The 6MWT will be performed using a 10 m track with cones demarcating the turning points [114]. Participants will be given verbal and visual instructions (physiotherapist will demonstrate one lap) before testing. Participants will be instructed to “Walk as far as possible without running in six minutes, completing as many laps as you can. Make sure you walk around the cones”. Participants will be instructed not to talk throughout the test. Participants will be given verbal encouragement, and every 30 s will be advised of the distance covered (in laps) and the time remaining. Distance will be measured to the nearest five metre mark.

The 6MWT is responsive, valid and reliable in children with CP and adults with TBI [115–117]. No psychometric data exist for Australian children and adolescents with an ABI, however it is expected children with an ABI will perform similarly to children with CP.

#### High-Level Mobility Assessment Tool (HiMAT)

The HiMAT was specifically developed to measure high-level motor performance in adults with TBI [118, 119]. This uni-dimensional measure consists of 13 items that assess high-level walking tasks, the ability to negotiate stairs, run, skip, hop and bound. This tool is used in both clinical and research settings. The test has reported high inter-rater (ICC = 0.99), test-retest reliability (ICC = 0.99, MDC = −2 to +4) and internal consistency (Cronbach alpha = 0.97) in adults with moderate and severe TBI [120]. Concurrent validity was established with the motor subsection of the Functional Independence Measure instrument and the gross function component of the Rivermead Motor Assessment, demonstrating moderate (r = 0.53, p < 0.001) and strong correlations (r = 0.87, p < 0.001), respectively [121]. Psychometric properties, including inter-rater reliability (ICC = 0.99), intra-rater reliability (ICC = 0.95, MDC = −3 to +4), responsiveness (area under the curve = 0.86) and validity (significant negative association with the Rivermead Post Concussion Symptoms Questionnaire, rho = −0.63, *p* < 0.001) of the HiMAT have been established in an adult population with mild TBI [122].

The HiMAT will be performed according to published recommendations [119]. These guidelines state that testing should take place along a flat, straight corridor where the middle 10 m of a 20 m track is timed except for the bounding and stair items. Two cones will be placed at either end of a 20 m track and video recorders will be set up at the 5 and 15 m interval. Markers will be moved to the 5 and 15 m interval for hopping as participants are only required to hop 10 m. For bounding items, a tape measure will be placed flat on the floor, and distance achieved will be measured to the nearest 0.5 cm. For ascending and descending stairs, cameras will be set-up at the top and bottom of the stairs (total of 10 stairs, scores adjusted accordingly for 14 stairs). A rail will be available on the right-hand side, and a wall on the left going up the stairs. If a participant is not comfortable ascending or descending stairs using the wall, participants can hold the physiotherapist’s hand, with the physiotherapist remaining behind the participant at all times. Participants will be given verbal instructions and one visual demonstration for all items. Participants will be instructed to perform at their maximum safe speed for items along the 20 m track and will be allowed one practice trial for each item before testing. Participants will be unaware that only the middle 10 m will be timed.

Raw scores measured in time and distance (centimetres) will be recorded and converted to a score from 0 to 4 where 0 represents the inability to perform an item, and scores from 1 to 4 represented increasing levels of ability (for both timing methods). The maximum score on the HiMAT is 54 (13 items with a maximum converted score of 4, plus one additional point on each stair item). Timed items in the HiMAT will be collected using a hand held stopwatch and a video timing method utilizing two video recorders (except bounding items).

Video cameras will be synchronised after the HiMAT has been completed with an auditory output from the assessor to start each HiMAT component. The 10 m time will be calculated from when the participant’s LL crossed the 5 and 15 m mark (as a ratio of distance and time). Cameras will be placed at the top and bottom of stair items and cameras will be synchronised again with an auditory output from the assessor to start. Time will be recorded from when the participant’s foot to clears the ground to when both feet are flat on the ground (ascending or descending stairs). Video recordings will be analysed using the video editing software program (Cyberlink powerdirector 9.0, New Taipei City, Taiwan) which de-interlaces video files to 25 images per second.

#### The Timed Up and Go (TUG) test

The Timed “Up & Go” (TUG) test is commonly used to quantify functional mobility and balance [123]. The TUG will follow original published guidelines with minor variations [124]. A chair with a backrest but without arm rests will be used and the height of the chair will be selected so that the participant’s feet are flat on the floor and hips and knees flexed to 90°. During assessment participants will be instructed on the word “go” to stand up (without using hands) and walk a distance of three metres, turn around a cone, walk back to the chair and sit down as fast but as safely as possible without running. Verbal and visual instructions and an unrecorded practice trial will be provided. Time will be recorded as participants leave and return to the chair. Children will perform three trials and the average will be recorded. If participants run or deviate from the testing protocol, they will perform an additional trial.

Participants will be timed using a handheld stopwatch and a video timing method. The video timing method involves placing a camera perpendicular to the chair so it captures time taken for the participant’s ischial tuberosity to clear the chair and on return, for the length of their posterior thigh to make full contact with the chair when sitting back down. Video recording will be analysed using the same software program as for the HiMAT (Cyberlink powerdirector 9.0, New Taipei City, Taiwan).

The TUG test was found to be reliable in Israeli children aged 7–14 years with TBI (ICC = 0.86) [125]. Israeli children at least one year post-severe TBI were found to perform significantly slower than typically developing peers on the TUG test. The TUG test is also a valid and reliable measure in children with CP. The TUG test was able to discriminate between levels I to III on the GMFCS (*p* < 0.001). The TUG test also demonstrated a moderately negative correlation with the Gross Motor Function Measure (GMFM) (*rho* = −0.524, *p* = 0.012) with lower TUG scores being associated with higher percentages of GMFM scores for the standing and walking dimensions. Finally, the TUG test has evidence of good intra-rater and test-retest reliability (ICC = 0.98; 95 % CI = 0.97-0.99 and ICC = 0.83, 95 % CI = 0.77–0.88, respectively) in children with physical disabilities [126]. Standard Error of Measurement was on average, 0.5 s [126].

### Activity performance domain

#### Assisting Hand Assessment (AHA)

The AHA is a Rasch analysed measure that evaluates how a child with a unilateral impairment (congenital hemiplegia or obstetric brachial plexus palsy) effectively makes use of his/her impaired UL in bimanual tasks [127]. The assessment is videotaped and 22 items are scored on a four point rating scale, with a range of possible raw scores of 22 to 88. Raw scores will be converted to interval level AHA units (logits- log odds probability units on a scale of 0 to 100), as recommended by test developers as the preferred method of reporting and measuring change [128]. Depending on the age of the child and in accordance with recommendations from test developers, one of the three versions of the AHA will be used (the small kids, school kids or adolescent version). The small kids and school kids versions have high test re-test reliability (ICC = 0.99 and 0.98 respectively) and reliability between the two forms is very high (ICC = 0.99). Reliability between the school kids and Ice Rocks (adolescent version) has not yet been reported, however the adolescent version will be used in this study for youth over the age of 12 years, as the school kids version is not valid nor age appropriate for this population. The AHA has been shown to be responsive to change due to UL interventions [103, 129, 130]. The MDC is estimated as five AHA units. Although the AHA is not validated in children with an ABI, it is expected that they will perform similarly to children with a unilateral impairment. In accordance with test recommendations, all therapists administering the AHA will have undergone standardised training and certification [127] and will be scored by one certified rater masked to group allocation and order of assessment.

#### Habitual Physical Activity (HPA)

HPA will be measured using ActiGraph® GT3X+ triaxial accelerometer (Shalimar, FL). The ActiGraph measures and records time varying accelerations of the trunk that ranges in magnitude from ± 6g. The ActiGraph accelerometer is a valid and reliable measure of ambulatory physical activity in children and adolescents with CP [57, 131]. ActiGraph-based estimates of Metabolic Equivalents (METS) is a valid method to differentiate between varying intensities of walking (slow, comfortable and brisk paced) in children and adolescents with CP using previously published cut points for TDC [131]. The ActiGraph has good to excellent reliability with stepping tasks (ICC = 0.66), light and moderate walking (ICC = 0.8) and vigorous walking (ICC = 0.7) in independently ambulant children and adolescents (aged 8 to 16 years) with CP [57]. In community dwelling adults with an ABI, ActiGraph-based estimates when compared to portable indirect calorimetry (Cosmed K4b^2^) as a criterion measure, provided a valid index of activity across different levels of walking intensities [132]. It is estimated that between four and five days of monitoring are required to reliably estimate daily physical activity in TDC [133]. Both weekdays and weekends will be included in the monitoring period due to marked differences in physical activity behaviour.

ActiGraphs will be fitted during assessment (on the dominant hip) and worn during waking hours for four days following assessment and training days. After four days it will be returned by courier for data extraction and analysis using the manufacturers ActiLife software. An activity log completed by the parents/guardians coupled with an ActiGraph to validate the ActiGraph accelerations against reported wear time will be given. Raw acceleration data will be considered for analysis where accelerations are recorded for >4h per day on at least one weekend and one weekday.

#### Canadian Occupational Performance Measure (COPM)

The COPM will be used to measure self-perception of performance and satisfaction with performance of individualised occupational goals [54, 134]. One occupational therapist will administer the COPM with the child/adolescent and parent at the baseline assessment. Caregivers and children will identify areas of difficulty in everyday occupational performance and prioritise these on a scale of 1 to 10. Three to five priority goals will be identified and perceived level of performance and satisfaction with performance will be rated on a scale between 1 and 10 (1 indicates poor performance/low satisfaction and 10 indicates very good performance/high satisfaction). An average score for performance and satisfaction is calculated [93]. Ratings from caregivers will be used for children under the age of eight years of age, and ratings from children over eight years of age in collaboration with their caregiver will be reported. The COPM was designed for all ages and disability groups, has good evidence of construct, content and criterion validity and good test re-test reliability for performance and satisfaction scores (ICC 0.76–0.89) [134–136]. The COPM is responsive to change in paediatric clinical trials [137, 138]. A change of two points or greater has been reported as being clinically significant [54].

#### Physical capacity and performance test-retest reproducibility and validity

Participants will have the option to participate in test-retest reproducibility study of rep_max_ of functional strength exercises, 6MWT, HiMAT and TUG test over two days at the testing facility during baseline (for the immediate group) or 20 week follow-up assessments (for the waitlist group). Accuracy between hand-timed and videos scores during the HiMAT and TUG test will also be investigated. Testing will be performed in the same order by the same physiotherapist.

Participants will also have the option to participate in the validation for the ActiGraph accelerometer as a measure of physical activity intensity in children and adolescents with an ABI. To validate the ActiGraph accelerometer, participants will be asked to return for one assessment day after they have completed the primary end-point (20 week assessment) for the RCT. For the validity protocol, participants will be asked to rest for a 5–10 min period and then conduct selected light, moderate and vigorous assessment tasks, interspersed with 5–10 min rest periods in a standardised manner. To finish, they will complete a 15 min Mitii™ training program adjusted for their individual capacity. These tasks will be performed whilst wearing an ActiGraph accelerometer, heart rate monitor and a portable indirect calorimeter (MetaMax®). For test-retest reproducibility of the ActiGraph accelerometer, participants will rest for five minutes before conducting selected light, moderate and vigorous assessment tasks, interspersed with 5 min rest period in a standardised manner over the baseline assessment and training days.

#### MobQues47

Activity limitations will be measured using the MobQues.[59] The MobQues was originally designed to measure the mobility limitations a child with CP experiences in everyday life and to cover a broad range of severity of mobility limitations, as rated by their parents. Two versions of the MobQues are available; the MobQues47 and the MobQues28 with 47 and 28 mobility items, respectively. Response options for the MobQues are: Impossible without help (score 0), very difficult (score 1), somewhat difficult (score 2), slightly difficult (score 3) or not difficult at all (score 4). Total scores are calculated by adding up the item scores. Total item scores are then divided by the maximum possible score (i.e. 188 for MobQues47 and 112 for the MobQues28) and multiplied by 100 to obtain values on a scale of 0 to 100 (with lower scores representing more severe mobility limitations). For research purposes, the shorter version (MobQues28) is recommended as it has stronger psychometric properties (based on Rasch analysis) whereas; the MobQues47 can be used for clinical applications, to assess the child’s limitations in a wide variety of mobility activities [59]. Content validity has been demonstrated as 46 out of the 47 test questions related to ‘mobility’ according the definitions in the ICF. Construct validity was demonstrated as MobQues scores decreased with increasing GMFCS levels (*p* < 0.001). In a subgroup of 162 children, MobQues score was positively correlated to GMFM-66 (Mobques47, *r =* 0.75; MobQues28, *r =* 0.67, *p* < 0.001) [139]. Reliability has also been demonstrated. For inter-rater reliability, high ICC were found for the MobQues47 (ICC = 0.92) and MobQues28 (ICC = 0.87). The SEM was 7.8 and 8.9 respectively. Intra-rater was higher with ICC of 0.96–0.99 and lower SEM (3.5–4.9) for both versions of the test [59]. The English version has not yet been cross-validated therefore, the results demonstrated may differ slightly to that in an English speaking population. The MobQues47 will be collected at baseline (to allow cross-validation) and in follow-up assessments, the MobQues28 will be used (stronger psychometric properties). This questionnaire will be available for caregivers to fill out in either paper or online format.

### Participation domain

#### The Child and Adolescent Scale of Environment (CASE)

The CASE was initially developed as part of the Child and Family Follow-Up Survey (CFFS) to monitor outcomes and needs of children with an ABI (as informed by the ICF) [140]. The CASE consists of 18 items on a three point ordinal scale (no, little or big problem) that asks parents about the impact of problems experienced with physical, social and attitudinal environment features of the child’s home, social and community and those related to the quality and availability of services of assistance that the child receives or might need. The CASE can be administered in five minutes and no specialist training is needed to administer it. The CASE has reported evidence for test re-test reliability (ICC = 0.75), internal consistency (α = 0.84–0.91) and construct/discriminant validity. Higher CASE scores (greater extent of environmental problem) were significantly associated with lower scores on the Child Adolescent Scale of Participation (CASP, more restricted participation, r = −0.57, [141]; r = −0.43, [142]) and on the Paediatric Evaluation of Disability Index (PEDI, more limited functional skills) mobility (r = −0.28) and social function (r = −0.31) subscales [143]. This questionnaire will be available for parents and/or guardians to fill out in either paper or online format.

#### The Child and Adolescent Scale of Participation (CASP)

The CASP measures participation of children with an ABI in home, school and community activities compared to typically developing children of the same age as reported by parents or guardians [140, 142]. Like the CASE, the CASP was developed as part of the CFFS to monitor outcomes and needs of children with an ABI. The CASP consists of 20 ordinal scaled items in four subsections: 1) Home participation, 2) Community participation, 3) School participation, and 4) Home and community living activities. The 20 items are rated on a four-point scale: “Age Expected (Full participation),” “Somewhat Restricted,” “Very Restricted,” “Unable”. A “Not Applicable” response can be selected when the item reflects an activity that the child would not be expected to participate in due to age (e.g. work). The CASP also includes open-ended questions that ask about effective strategies and supports and barriers that affect participation. The CASP takes about 10 min to administer and no specific training is needed.

The CASP has reported evidence of test re-test reliability (ICC = 0.94), internal consistency (α ≥ 0.96) and construct and discriminant validity. Moderate correlations were found between the CASP scores and scores from measures of functional activity performance on the PEDI (r = 0.51–0.75), extent of child impairment on the Child and Adolescent Factors Inventory [CAFI (r = −0.58 to −0.66) and problems in the physical and social environment as measured by the CASE (r = −0.43 to −0.57) [140, 142, 143]. Significant differences in CASP scores were found related to type of disability [142]. Children without disabilities, on average, had significantly higher CASP scores than children with disabilities. No significant differences were found related to age category [142]. The CASP will be available for parents and/or guardians to fill out in paper or online format.

#### Participation and Environment Measure for Children and Youth (PEM-CY)

This parent-report instrument examines the frequency and level of participation in home, school and community settings [144]. In addition, the measure also addresses whether the surrounding environment makes it easier or harder to participate. There are 10 items in the home section, five in the school section and 10 in the community setting. For each item, the parent is asked to identify how frequently (over the past four months) the child has participated (eight options: daily to never); how involved the child typically is while participating (five point scale: very involved to minimally involved); and whether the parent would like to see the child’s participation in this type of activity change (no or yes, with five options for the type of change desired). After each section the parent is then asked to report on whether certain feature of the environmental makes it easier or harder for the child to participate. The PEM-CY has reported moderate to good internal consistency (0.59 and above) and test-retest reliability (0.58 and above) in a population of children (aged 5 to 17 years) with and without disabilities residing in the United States of America and Canada (*n* = 576) [144]. The PEM-CY will be collected at baseline using either paper or online questionnaire format to gain an understanding of the participation of children and adolescents and the impact of environmental support.

#### Strengths and Difficulties Questionnaire (SDQ)

The Strengths and Difficulties Questionnaire (SDQ) [145, 146] is a 33 item parent-rated questionnaire that is used to assess parents’ perceptions of pro-social and difficult behaviours in their child. Parents are required to respond to 25 questions about their child’s behaviour in the last six-months using a three-point likert scale (i.e., “0” = not true to “2” = certainly true). These 25 questions combine to create five sub-scales of: frequency of emotional symptoms; conduct problems; inattention/hyperactivity; peer problems; and prosocial behaviour (e.g. “considerate of other people’s feelings”). A total score for each scale (0–10) and overall total difficulties score (0–40) will be calculated, with higher scores indicating more distress on all scales except prosocial behaviour. Scores of 17 or above for the total difficulties scale will be used a clinical cut-off point. Scores from the five sub-scales and the overall difficulties scale will be used as a measure of the child’s psychological functioning. The overall total difficulties score has been demonstrated to have moderate to high internal consistency (α = 0.73–0.82) and test-retest reliability (*r* = 0.77–0.85) [147].

### Quality of Life

#### Kidscreen-52

The Kidscreen-52 is a generic measure of QOL which will be used to compare parents report of QOL for children with ABI compared to their age, gender and matched pair. The Kidscreen-52 has been described as the most useful generic measure of QOL of children as it addresses the multidimensional construct of QOL through various domains and focuses specifically on the well-being of children. It was developed to implement the views of children, through focus group work of 22,110 children [148]. The Kidscreen-52 Questionnaire takes 15–20 min to complete and consists of 52 questions across 10 domains: Physical Well-being; Psychological Well-being; Moods and Emotions; Self-Perception; Autonomy; Parent Relations and Home Life; Social Support and Peers; School Environment; Social Acceptance (Bullying); and Financial Resources. The items use a five point Likert-type scale to assess either the frequency of certain behaviours/feelings or, in a small number of cases, the intensity of attitude. Higher scores on the Kidscreen-52 indicate better health-related QOL and well-being. During development of this measure, a child self-report was administered for children 8–18 years old and a parent proxy version was administered for parents of children 5–18 years. Children below the age of eight were unable to self-report as their reports were likely to be unreliable. Reliability was calculated by Cronbach’s Alphas and ranged between 0.76 and 0.89 throughout the 10 domains of health-related QOL. Convergent and discriminant validity were tested using information of child’s physical and mental health. Correlations of up to 0.55 were found when correlating the Kidscreen-52 dimensions with frequency of physical complaints [148].

#### CHU-9D

The Child Health Utility (CHU-9D) will be used to measure participant QOL (parent/proxy completion). This relatively new instrument is specifically designed to measure health-related QOL in children and those affected by a disability from a young age [149]. It contains nine domains: worried, sad, pain, tired, annoyed, school/homework, sleep, daily routine and able to join activities. Each domain has five response levels. The CHU-9D is scored on a scale of 0–100 using preference weights which allow the calculation of Quality Adjusted Life years (QALYs) for use in economic evaluation. Preference weights are available from a general adult United Kingdom population and from an Australian population of 11–17 year old adolescents [150, 151].

### Environmental and personal factors

The same study questionnaire which was used for a previous Mitii™ protocol will be used [89]. This questionnaire was developed to capture important demographic information that has been shown in the literature to influence participation. Information includes family ethnicity, household income, socio-economic status, family structure and supports, and family interests. The questionnaire will be administered at baseline. A measure of social advantage/disadvantage will also be derived from postcode of residence using the Index of Relative Socio-economic Advantage/Disadvantage (2006) from the Australian Bureau of Statistics [152]. Deciles will be reported on a continuum with lower scores reflecting greater socio-economic disadvantage and higher scores reflecting socio-economic advantage.

### Exit interview

An exit interview will be conducted with participants and their parents or guardians to investigate engagement in web-based therapy in the home environment to guide future clinical implementation. Semi-structured interviews will be conducted by a therapist who is not involved in the conduct of the study, audio recorded and transcribed verbatim until saturation of data is achieved. Transcripts will be analysed using an inductive approach.

### Healthcare costs

Virtual trainers will keep a diary to document the amount of time taken to monitor and progress programs, contact families and troubleshoot any technical difficulties. These data will be used to estimate the costs associated of the Mitii™ program. A cost will be assigned to each resource; allied health visits by state health wage rates; medical care and diagnostic/investigational services according to the fees in the national Medicare Benefits Schedule [153].

## Statistical analysis

### Sample size

The primary basis for sample size calculation for this study is adequate power (80 %) to detect a clinically important difference for the comparison between the functional effects of Mitii™ (as measured on the AMPS and rep_max_ of functional strength exercises) and usual care at the primary endpoint (20 weeks). This study examines a continuous response variable from matched pairs, waitlist control and immediate-intervention participants with one waitlist control per immediate intervention participant.

In a previous study of Mitii™ in children with unilateral CP (n = 102) the response within each group was normally distributed with SD 0.40 on the AMPS process subscales [49]. To detect a real changes in ADL processing abilities (0.30 units of greater) [92] between groups with 80 % power and α = 0.05, 29 children from each group are required to complete 20 week follow-up assessments.

For rep_max_ of functional strength exercises in a previous reliability study, the response within each subject group was normally distributed with a SD of five sit-to-stand repetitions [93]. If the clinically important difference between groups is four repetitions we will need 26 children from each group to complete 20 week follow-up assessments with, α = 0.05 [47].

Based on a previous RCT using 3T fMRI there were activation in the representative cortex for motor studies with good signal to noise ratio. Participant numbers will allow for some loss of information due to participant refusal (10 %) and scans where motion is a confounder (10 %). To detect fMRI changes between baseline to follow-up assessment (20 weeks) with 80 % power and a SD of 0.65, we will require 39 participants. If the supplementary motor area (SMA) is considered, given coefficients of variation (COV) for control subjects performing motor tasks (COV of 11 % in PM1 and 35 % in SMA, and activation signal of 1.5 %, we will be able to detect differences in percentage activation levels over time as small as 0.47.

### Statistical analysis

Analysis will follow standard principles for RCTs. Primary and secondary outcomes will be assessed using two-group comparisons on all participants with evaluable data at 20 week follow-up assessments on an intention-to-treat basis. Statistical significance will be set at *p* < 0.05 for primary outcomes. Statistical significance for secondary outcomes will be defined separately for each suite of analyses prior to analyses being undertaken. Each significance level will be set to account for both type I and type II error rates due to the number of multiple comparisons. Validity of results will be checked using baseline and general descriptive information available for all eligible families. This includes comparing the key characteristics of families who completed the study with those who enrolled in the study but did not complete, and those who did not enrol. The primary outcome measures immediately post-intervention at 20 weeks will be AMPS and rep_max_ of functional strength exercises. Outcomes between treatment groups will be compared at follow-up using linear regression models, where treatment group (intervention or waitlist) and baseline score will be entered in as main effects. Secondary analyses will compare the outcomes between groups for physical activity, participation (CASE and CASP) and QOL (domains of Kidscreen-52).

For reproducibility studies (AMPS, physical activity capacity and performance measures and attention and executive functioning measures), units of variance will be assessed using an analysis of variance (ANOVA) model with Bland Altman Plots with 95 % limits of agreement (LOA). Intra-class correlation coefficients (model: 2-way mixed effects with absolute agreement) and 95 % confidence intervals will be calculated and interpreted as greater than 0.75 as excellent correlation, between 0.4 and 0.75 as adequate and less than 0.40, poor correlation [154]. The SEM will be calculated using [155]:

$$ SEM= Standard\  Deviation\ (SD) \times \sqrt{\Big(1-ICC}\Big) $$

The MDC will be calculated using [155]:

$$ MDC=SEM \times 1.96 \times \sqrt{2} $$

For attention and executive functioning measures, reliable change indexes (RCI) will be calculated using [156]:

$$ RCI=\left( retestscore - initialscore\right)\div \left( standard\  error\  of\  difference,\  SDiff\right) $$

Validity of the ActiGraph accelerometer using oxygen uptake (*V*O_2_) as the criterion measure will be assessed using one-way repeated-measures ANOVA with post-hoc pair-wise comparisons with Bonferroni corrections for multiple comparisons.

### Economic analysis

A within trial cost-utility analysis will be conducted to compare the Mitii™ intervention to usual care from both a health care and societal perspective. The costs and outcomes for each group will be compared using incremental cost-effectiveness ratios (ICERs) (i.e. [cost Mitii™ – cost waitlist/ [QALYs Mitii™ – QALYs waitlist].

The CHU-9D will be scored using the Australian preference weights and total QALYs estimated for the period of the trial [151]. Key variables (e.g. cost of delivering the intervention, scoring the CHU-9D with the UK preference weights) will be altered and tested in one-way and multi-way sensitivity analyses to generate a range of possible ICERs for the intervention. Economic data will be bootstrapped to generate 95 % CI.

### MRI analysis

#### Structural parcellation

Cortical reconstruction and volumetric segmentation will be performed using the MPRAGE images with the Freesurfer image analysis suite (http://surfer.nmr.mgh.harvard.edu). Briefly, non-brain tissue will be removed using a hybrid watershed/surface deformation procedure [157]. Subcortical white matter and deep grey matter volumetric structures will be segmented automatically and intensity inhomogeneity of the images will be corrected [158, 159]. The cerebral cortex will be parcellated into 34 units per hemisphere based on gyral and sulcal structure [160, 161]. In addition to cortical regions, the left and right thalamus, left and right cerebellum and brain stem will be extracted. The accuracy of the cortical and subcortical parcellation will be assessed visually. An axial slice will be manually defined below the inferior most slice on which the pons was visible to include only the most caudal part of the brain stem.

A termination mask will be generated to prevent diffusion tractography streamlines from crossing the cortical folds as described previously [162]. Briefly, the interface between white matter and grey matter will be identified and this boundary shifted one voxel into the grey matter. Streamlines will be terminated when penetrating more than one voxel deep into grey matter.

#### Diffusion processing

An extensive pre-processing procedure will be followed to detect and correct for image artefacts caused by involuntary head motion, cardiac pulsation and image distortions [163]. In brief, images with within-volume movement will be detected using the discontinuity index and excluded from further analysis [164]. Image distortions caused by susceptibility in homogeneities will be reduced using the field map, employing FUGUE and PRELUDE tools available with FSL and intensity in homogeneities will be removed using n3 correction [165, 159]. Subsequently, signal intensity outlier voxels (caused by cardiac pulsation, head motion and other artefacts) will be detected and replaced using DROP-R [166]. DROP-R will be modified from the originally proposed method to employ a higher order model of the diffusion signal suitable for the detection and replacement of outliers in high b-value diffusion data (HOMOR) [167]. Between-volume registration to account for head movement during the scan time will be performed using FMAM with adjustment of the b-matrix [168–170] . Following these steps, fractional anisotrophy (FA) will be estimated from the corrected diffusion data. Constrained spherical deconvolution (http://nitrc.org/projects/mrtrix) will be employed to estimate the fibre orientation distribution for tractography at maximum harmonic order [85, 171].

#### Connectome construction

Diffusion and structural data will be co-registered using a rigid-body transformation with FLIRT (part of FSL) by registering the FA to the skull-stripped MPRAGE [165]. Registration accuracy will be checked visually. Five million probabilistic streamlines will be generated, seeding throughout the entire brain volume, to produce a whole brain tractogram. Streamlines will be prevented from crossing cortical folds by applying the termination mask generated from structural data (see section Structural parcellation). Cortico-cortical, cortico-thalamic, cortico-cerebellar and cerebello-cerebellar connections will be extracted from the whole brain tractogram by hit-testing both terminal end points of every streamline with every cortical and cerebellar region. For brain stem connections, only one terminal endpoint will be required to reside within the cortical, thalamic or cerebellar region, with any part of the streamline passing through the brain stem. For every possible link between any pair of nodes, the number of connecting streamlines will be noted. Median FA values will be calculated by sampling the diffusion maps at every step of the selected streamlines. Connections with fewer than 250 streamlines (average) in children with typical development will be excluded from further analysis (threshold determined empirically). Results will be recorded in enriched connectivity matrices.

#### Statistical analysis of the brain network

Statistical analysis of the brain network will be performed using the network based statistic toolbox for Matlab (https://sites.google.com/site/bctnet/comparison/nbs) [172]. A general linear model will be used to identify differences in FA between participant groups for every connection, using age as a confounding variable.

## Discussion

This proposed study presents the background and design for a matched pairs, randomised waitlist controlled trial comparing 20 weeks of intensive Mitii™ training to usual care for children with an ABI. This study is the first to investigate the effects of a multi-modal training program delivered over the internet compared to usual care and will also be the largest of its type in this population. Furthermore, we will be evaluating outcomes of the Mitii™ training program across all domains of the ICF using the most valid and reliable assessment tools available for use. We anticipate that the results of this study will be disseminated through peer reviewed journals and national and international academic conferences.

## Additional files

Additional file 1:
**Mitii** ^**TM**^ **ABI participant manual.ᅟ**
Additional file 2:
**Mitii** ^**TM**^ **ABI rewards chart (child version).ᅟ**
Additional file 3:
**Mitii** ^**TM**^ **ABI modules.ᅟ**

## Abbreviations

ABI: Acquired brain injury
ADHD: Attention deficit hyperactivity disorder
ADL: Activities of daily living
AHA: Assisting hand assessment
AMPS: Assessment of motor and process skills
AROC: Australasian rehabilitation outcome centre
BGBS: Basal ganglia and brainstem score
BIM: Bimanual training
BM: Brain maldevelopment
BMI: Body mass index
BoNT-A: Botulinum toxin serotype A
BOT-MP: Bruininks-oseretsky test of motor proficiency
BRI: Behavioural regulation index
BRIEF: Brief rating inventory of executive functioning
CAI: Centre for advanced imaging
CASE: Child and adolescent scale of environment
CASP: Child and adolescent scale of participation
CDGM: Cortical and deep grey matter
CHU-9D: Child health utility 9D
CI: Confidence interval
COPM: Canadian occupational performance measure
CP: Cerebral palsy
CSD: Constrained spherical deconvolution
CTMT: Comprehensive trail making test
D-KEFS: Delis-kaplan executive function system
dMRI: Diffuse magnetic resonance imaging
DT: Duel task
EPI: Echo-planar imaging
ET: Echo time
FC: Functional connectivity
fMRI: Functional magnetic resonance imaging
FOV: Field of view
FSIQ: Full scale intelligence quotient
GCS: Glasgow coma scale
GEC: Global executive composite
GEEs: Generalised estimating equations
GMFCS: Gross motor functional classification system
GMFM: Gross motor function measure
GRE: Gradient-recalled-echo
GS: Global score
HiMAT: High-level mobility assessment tool
HPA: Habitual physical activity
HS: Hemispheric score
ICC: Intraclass correlation coefficients
ICERs: Incremental cost-effectiveness ratios
ICF: International classification of functioning, disability and health
IQ: Intelligence quotient
JTTHF: Jebsen taylor test of hand function
KM: Krägeloh-mann
LI: Laterality index
LOA: Limits of agreement
LOC: Loss of consciousness
MACS: Manual ability classification system
MCI: Metacognition index
mCIMT: Modified constraint induced movement therapy
MDC: Minimum detectable change
METS: Metabolic equivalents
Mitii™: Move it to improve it
MUUL: Melbourne assessment of unilateral upper limb function
MRI: Magnetic resonance imaging
M2PD: Moving two point discrimination
PEDI: Pediatric evaluation of disability inventory
PEM-CY: Participation and environment measure for children and youth
PRI: Perceptual reasoning index
PSI: Processing speed index
PTA: Post-traumatic amnesia
PWM: Periventricular white matter
QALYs: Quality adjusted life years
QCPRRC: Queensland cerebral palsy and rehabilitation research centre
QOL: Quality of life
QPRS: Queensland paediatric rehabilitation service
RCT: Randomised controlled trial
Rep_max_: Repetition maximum
ROM: Range of motion
RT: Repetition time
SD: Standard deviation
SDQ: Strengths and difficulties questionnaire
SEM: Standard error of measurement
sMRI: Structural magnetic resonance imaging
TEA-Ch: Test of everyday attention for children
TBI: Traumatic brain injury
TDC: Typically developing children
TMS: Transcranial magnetic stimulation
TOL: Tower of london
TUG: Timed up and go
TVPS: Test of visual perceptual skills
UL: Upper limb
VCI: Verbal comprehension index
VR: Virtual reality
WeeFIM: Functional independence measure for children
WHO: World health organisation
WISC-IV: Wechsler intelligence scale for children–fourth edition
WM: Working memory
3T: 3 tesla
6MWT: 6-minute walk test
