# Supplementary material for: Mitii™ ABI: study protocol of a randomised controlled trial of a web-based multi-modal training program for children and adolescents with an Acquired Brain Injury (ABI)
Source: BMC Neurol. 2015 Aug 19;15:140. doi: 10.1186/s12883-015-0381-6 (PMC4544804; doi:10.1186/s12883-015-0381-6)
Supplement: Additional file 1: — Mitii TM ABI participant manual.ᅟ [file 12883_2015_381_MOESM1_ESM.pdf]

Queensland cerebral palsy & rehabilitation research centre

Mitii™

Move it to improve it

Participant Training Manual

Queensland Cerebral Palsy & Rehabilitation Research Centre

Level 7, Block 6, RBWH, Herston QLD 4029 Australia
Study Telephone 07 3636 5361 • Facsimile 07 3636 5538
Email qcprrc@uq.edu.au.au • QCPRRC Phone 07 3636 5542

Getting Started

Before logging into Mitii you will need the following:

1. **Computer or laptop that is connected to the internet**
   Ideally plug the computer directly into the internet by connecting the network cable to the computer, otherwise wireless internet will work – but it might be a little slower to load the programs.
2. **Create a Kinect Friendly Zone**
   The Mitii program is very sensitive to objects and other people who are around. See instructions and photo below.
3. **Plug in Kinect to your computer and power point**
   Kinect should be placed near the edge on a flat surface just below your computer screen.
4. **Install the Mitii program on your computer**
   See instructions below.

Once you have Mitii installed on your computer and set up your training area you will need to log into the program. Connect to the internet and ensure the Kinect is plugged into both the computer and wall. Next, double click on the Mitii logo on your desktop.

Use your username and password to log into the program.

Customer: Queensland University

My Username is: ______________________________

My Password is: ______________________________

This is a generic password which has been pre-set. You can change this password once you have logged on the Mitii program.

The program should remember your customer name and username next time you log on but you will have to remember your password! If you forget, simply email us and we will reset your password again.

Creating a Kinect Friendly Zone

- Place the sensor near the edge on a flat, stable surface.
- Position the sensor between 1/2 metre and 2 metres from the floor. Ideally, the sensor should be just below your computer screen.
- Avoid positioning the sensor in direct sunlight.
- Do not manually tilt the sensor; it adjusts automatically.
- Be careful not to drop the sensor.
- Clear the area between the sensor and yourself.
- Stand back 2 metres.
- Ensure there is about a 2 metre area free from objects or other people.

Kinect needs to see your entire body!

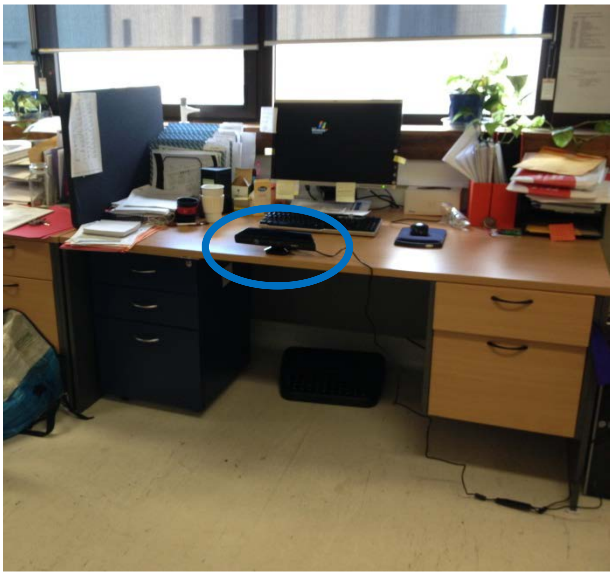

How NOT to set up your Kinect!

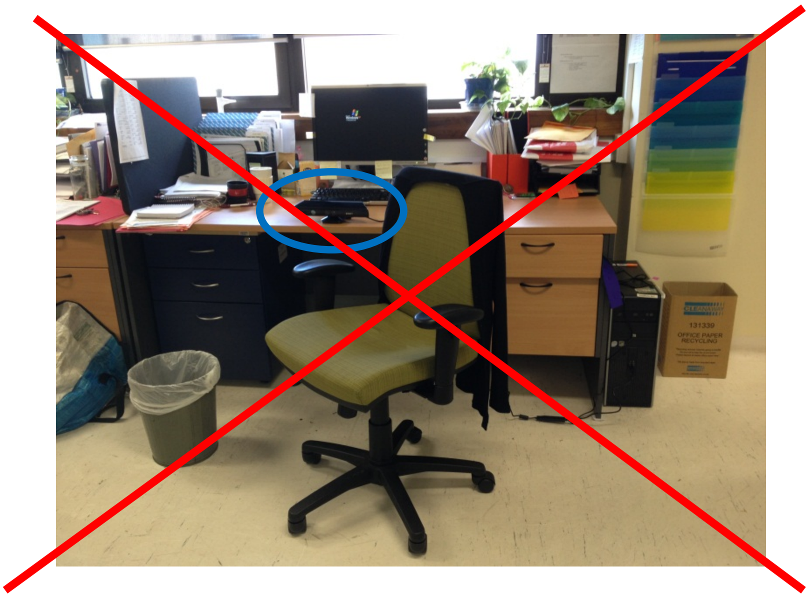

Connecting the Kinect to your computer

1. Plug Kinect into power point.

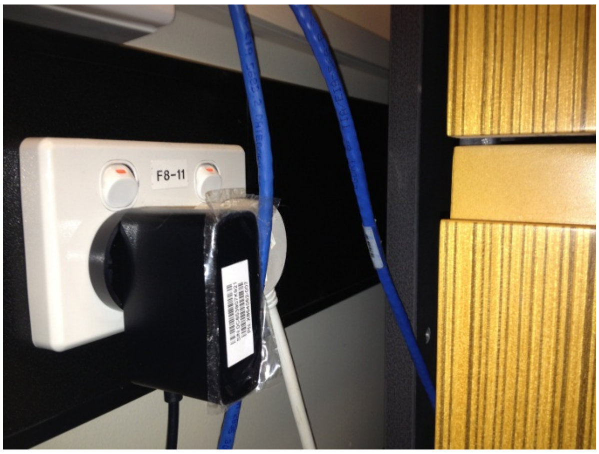

2. Plug the USB cable into a spare USB port.

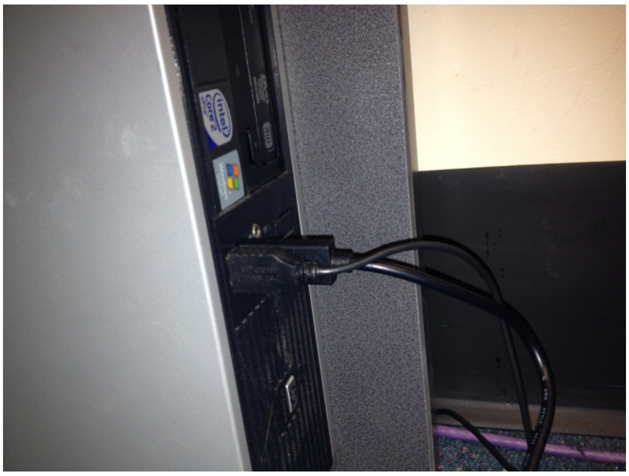

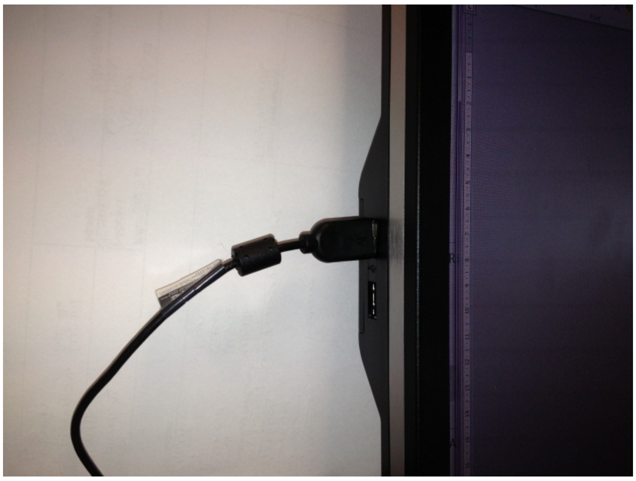

3. Kinect should then automatically install on your computer. Please contact us if this does not work.

How to install the Mitii program on your computer

*****Please note depending on the security preferences on your computer you may be prompted with a few additional steps which you will need to navigate through*****

1. Ensure your computer or laptop (Windows computer only! Mac computers do not support the software) is turned on and connected to the internet.

2. Open up **Internet Explorer** from the Start Menu located in the bottom left hand corner. The program will ONLY download in Internet Explorer, so check that you have not selected Mozilla Firefox or another internet browser.

3. Once you have opened up **Internet Explorer** type the following into the top panel of your browser: https://mitiistorage.blob.core.windows.net/vsdeploy/clickonce/publish.htm We will also email this link to you, so you can click on the link if you would prefer.

4. The page you will be directed to can be seen below. Click on **INSTALL**. If the yellow box at the bottom appears, click **RUN**.

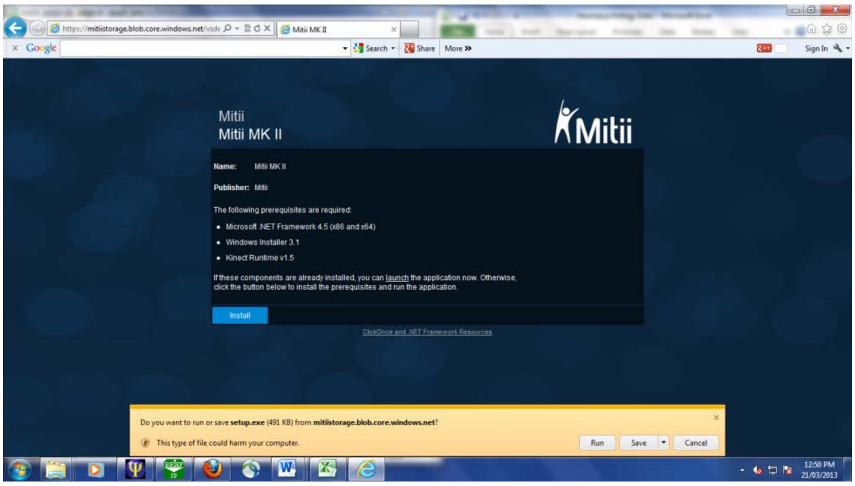

5. You will then be asked to install Kinect Runtime v1.5. Click **INSTALL**.

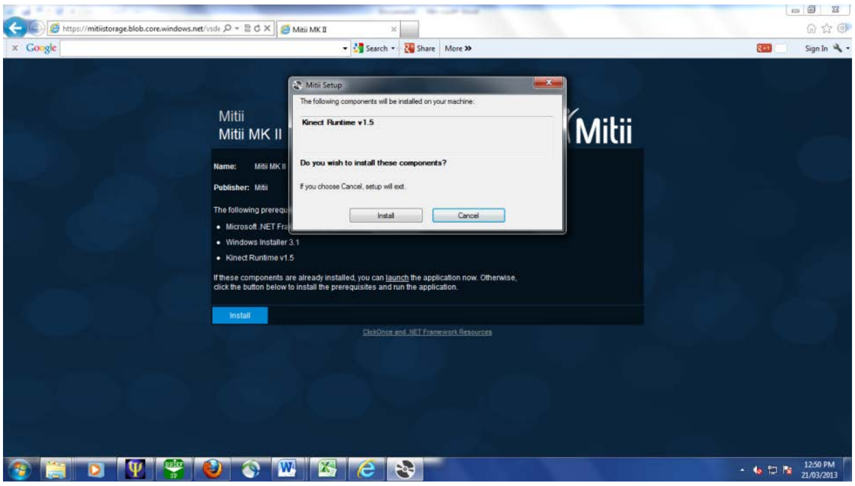

6. Kinect Runtime v1.5 will then start to install. This may take a few minutes.

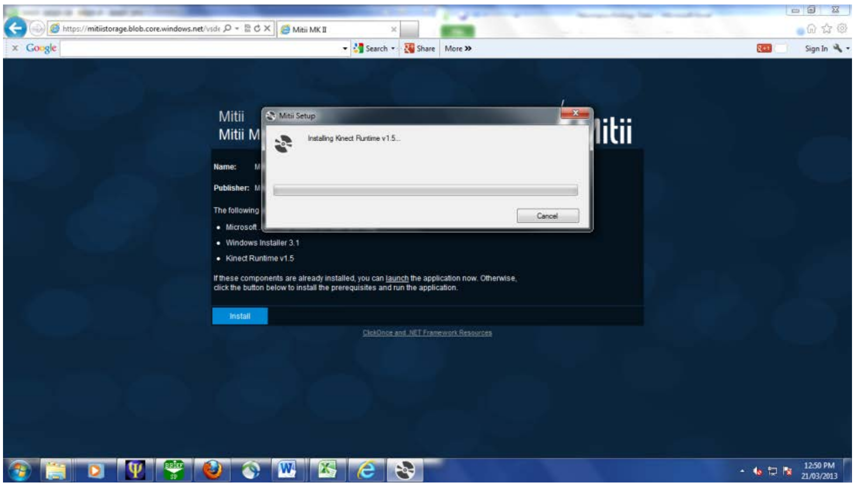

7. You will then need to **ACCEPT** the terms and conditions by clicking the empty box.

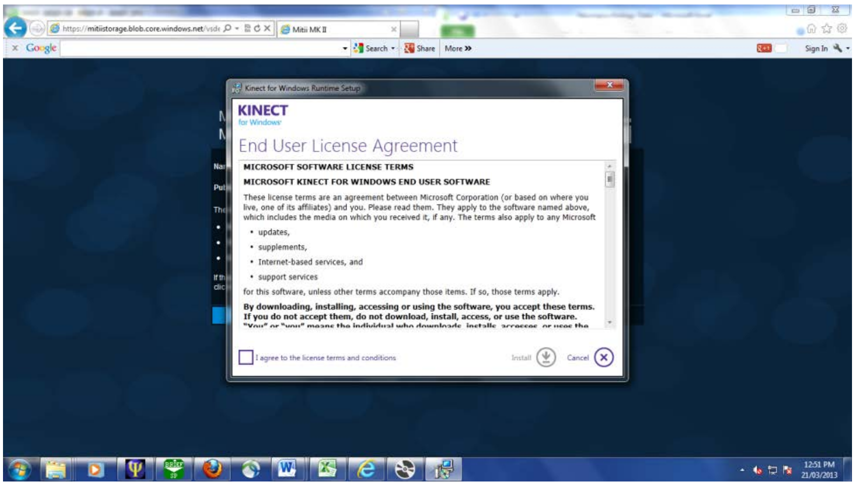

8. Once you have ticked the box, you then need to select **INSTALL**.

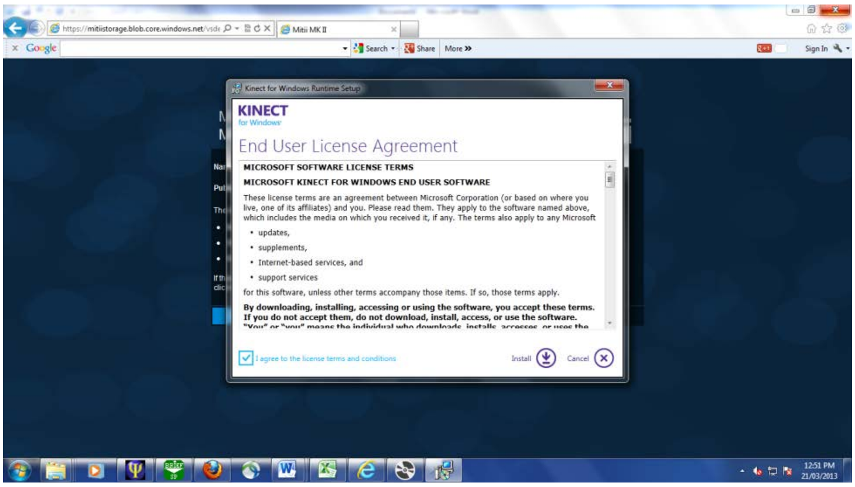

9. On the next screen select **CLOSE**.

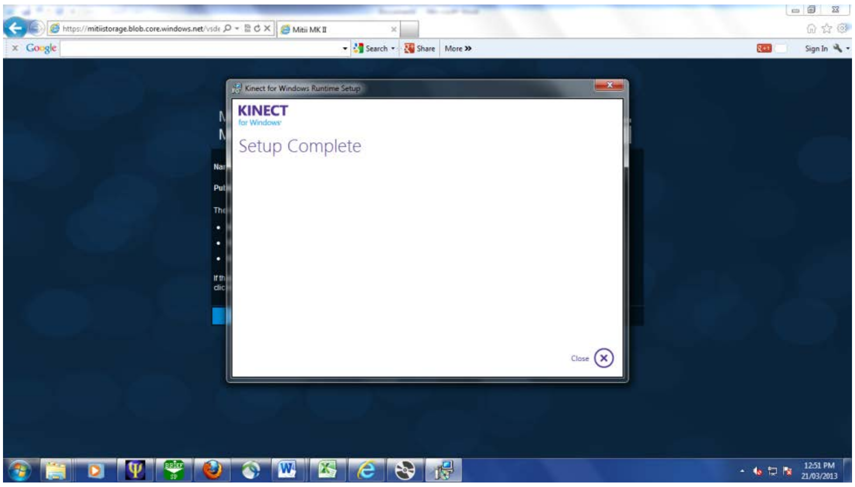

10. The application will then launch itself. This may take a few moments.

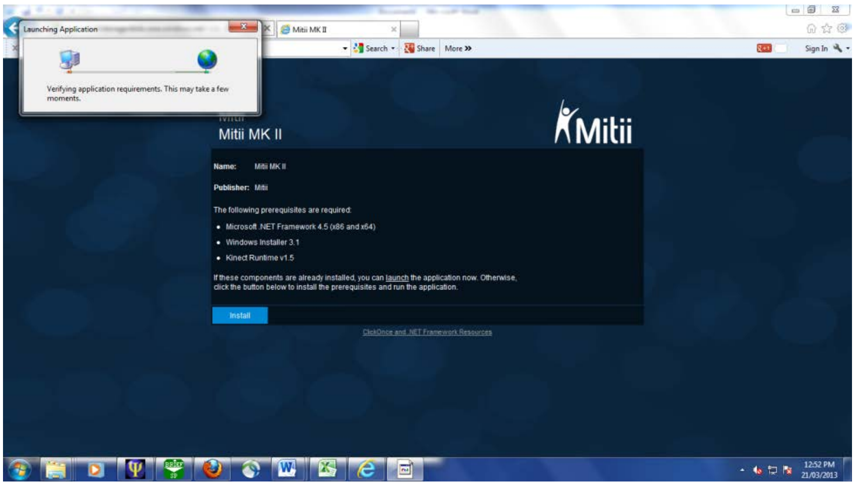

11. You will then be asked to install Mitii MK II. Click **INSTALL**.

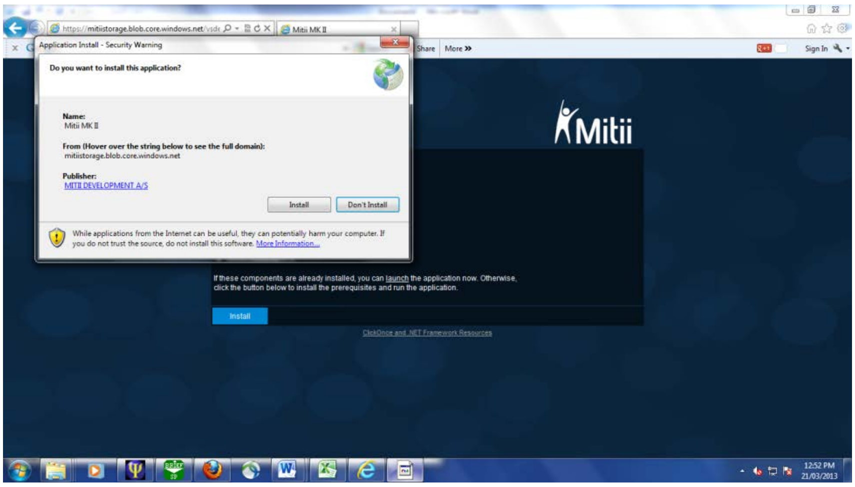

12. Mitii MK II will then begin to download. This may take a few minutes.

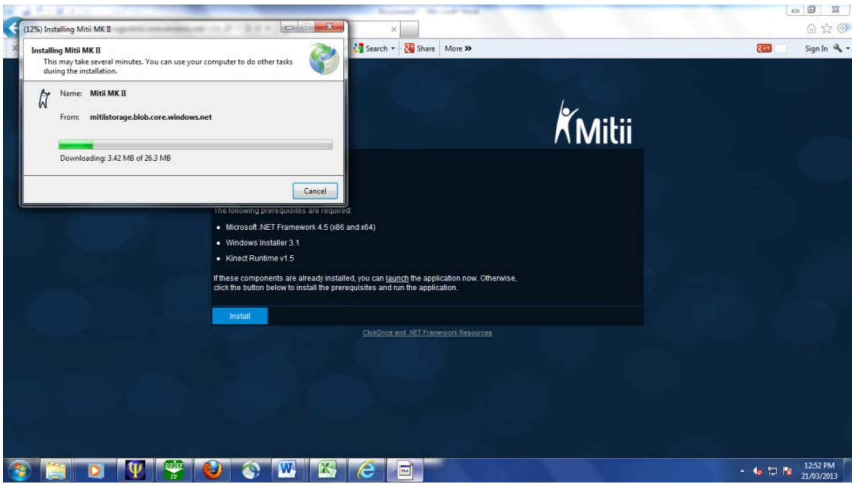

13. Once the program is installed you will be directed to the login screen.

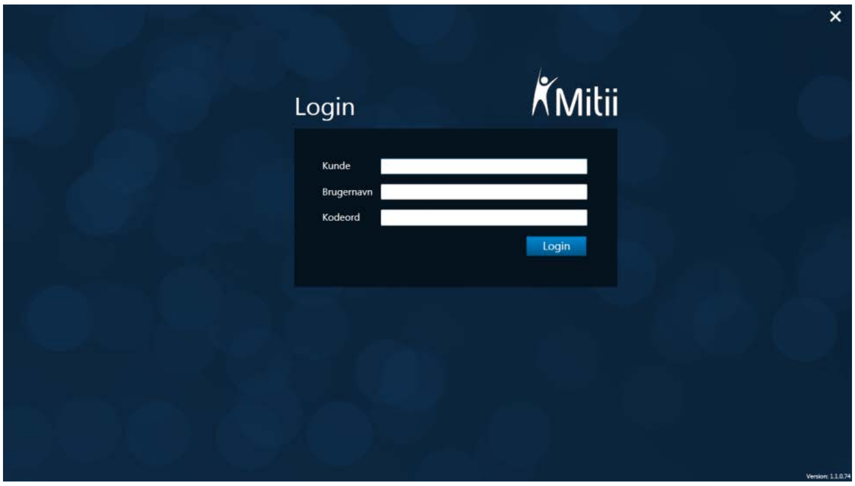

14. To login to Mitii you will need to enter your log in details. These have been provided to you during your training.

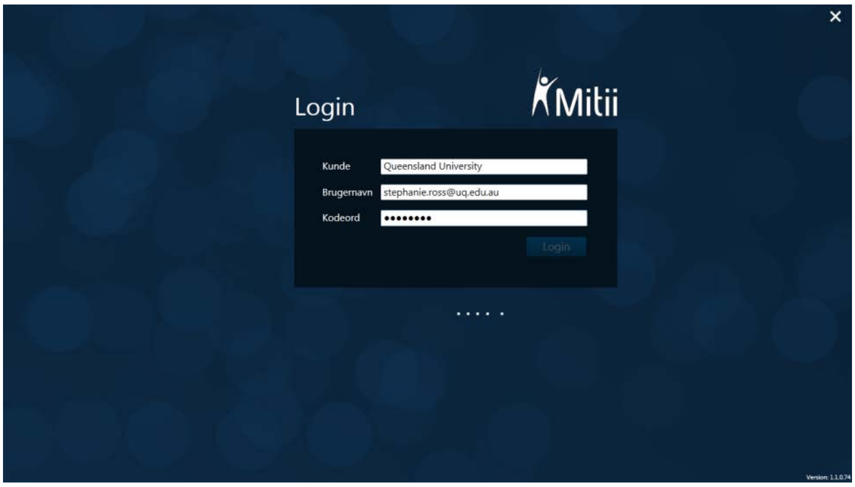

15. When you log in you will come to the screen below. If you would like to change your password select **CHANGE PASSWORD**.

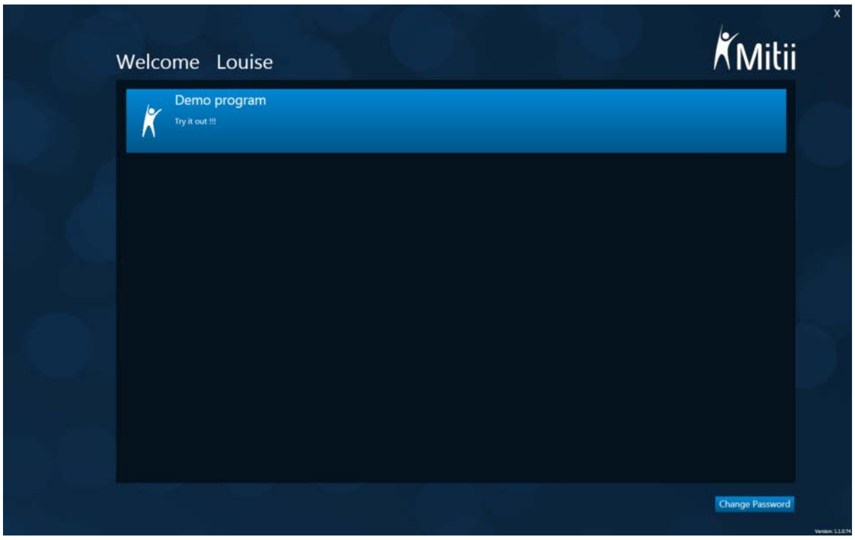

Your Week 1 program will be displayed and you are now ready to **BEGIN YOUR MITII TRAINING**!

Mitii Modules

There are multiple different Mitii modules which the team will select for you based on your needs (please see Additional File 3 for modules).

We will go through these in detail on the training day.

The background and the pictures may change and the order they come in, however the modules remain the same.

There will be an instruction video before each module to help you learn the module. As you become more familiar with Mitii we will remove these videos unless we have specific instructions to give you.

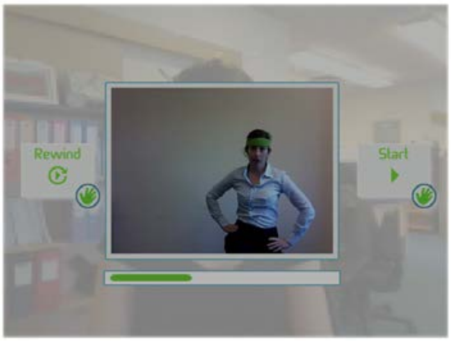

Challenging you over the whole program

We will be in contact with you by email (approximately once a week) to let you know when we have changed your program and tell you which modules we want you to add a step block or foam to make it harder.

For any game you may be asked to:

Lunge (one leg in front of the other)

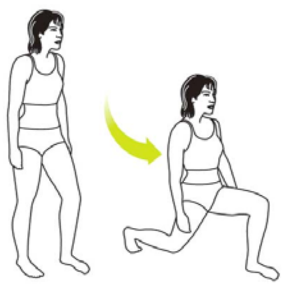

Sit to stand on and off a chair

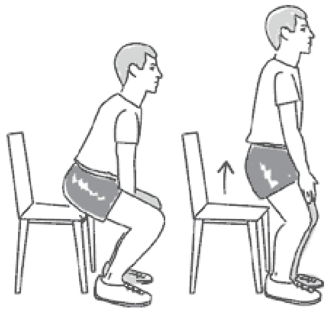

Step up onto the step

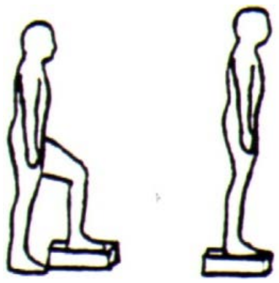

Squat down and either stand or jump up

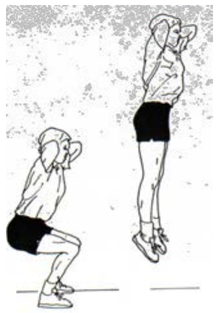

Step up sideways onto the step

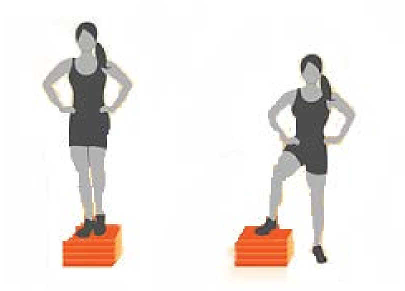

Or stand on the foam and balance

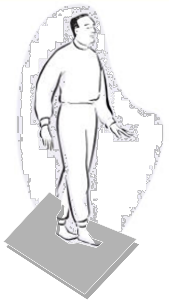

Have fun!
