# Supplementary material for: Mitii™ ABI: study protocol of a randomised controlled trial of a web-based multi-modal training program for children and adolescents with an Acquired Brain Injury (ABI)
Source: BMC Neurol. 2015 Aug 19;15:140. doi: 10.1186/s12883-015-0381-6 (PMC4544804; doi:10.1186/s12883-015-0381-6)
Supplement: Additional file 2: — Mitii TM ABI rewards chart (child version).ᅟ [file 12883_2015_381_MOESM2_ESM.pdf]

Mitii Reward Chart

Make your way through the Mitii Reward Chart to get prizes along the way and special rewards at the end of each 5 week stage!

Practice **30** minutes of Mitti each day for one sticker. You will get a prize when you have 6 stickers in a week!

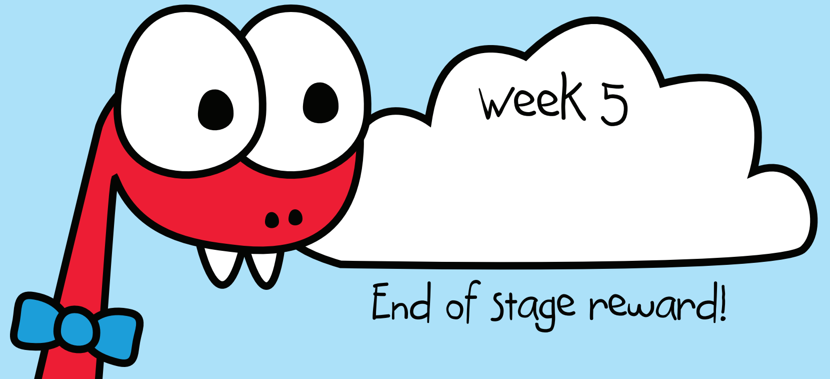

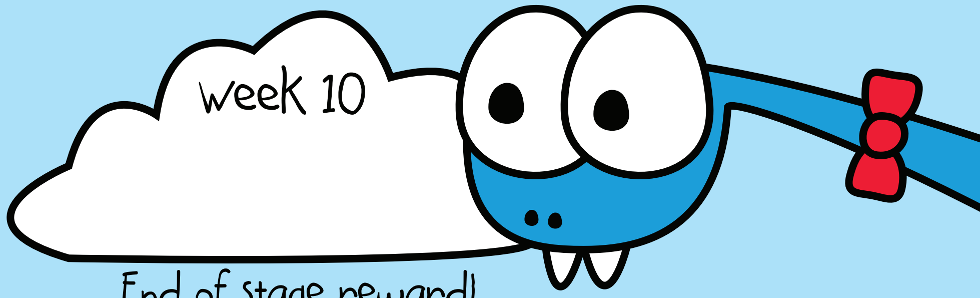

6 Prize Time!	Week 1 Prize	Week 2 Prize	Week 3 Prize	Week 4 Prize	Week 5 Prize	You have now finished Mitii Stage **1** (Well Done!)	Week 6 Prize	Week 7 Prize	Week 8 Prize	Week 9 Prize	Week 10 Prize	WELL DONE! You have now finished Mitii Stage **2**
5												
4												
3												
2												
1												
**Week**	1	2	3	4	5		6	7	8	9	10	

6 Prize Time!	Week 11 Prize	Week 12 Prize	Week 13 Prize	Week 14 Prize	Week 15 Prize	WELL DONE! You have now finished Mitii Stage **3**	Week 16 Prize	Week 17 Prize	Week 18 Prize	Week 19 Prize	Week 20 Prize	WELL DONE! You have now finished Mitii
5												
4												
3												
2												
1												
**Week**	11	12	13	14	15	Week 15 End of stage reward!	16	17	18	19	20	

Half way!

Week 20 End of stage reward!

© ClareVacha
