# Supplementary material for: Mitii™ ABI: study protocol of a randomised controlled trial of a web-based multi-modal training program for children and adolescents with an Acquired Brain Injury (ABI)
Source: BMC Neurol. 2015 Aug 19;15:140. doi: 10.1186/s12883-015-0381-6 (PMC4544804; doi:10.1186/s12883-015-0381-6)
Supplement: Additional file 3: — Mitii TM ABI modules.ᅟ [file 12883_2015_381_MOESM3_ESM.docx]

**Mitii^TM^ modules (Please note: Content of Mitii^TM^ is copyright to Mitii^TM^ Development A/S)**

| **Name** | **Part of your body to use** | **Description** | **Instructions** | **Domains trained** | **Sample Screen** |
| --- | --- | --- | --- | --- | --- |
| Match Two Images (2-hand exercise) | Both hands | Use both hands to drag the matching pictures into the circle | Find the matching pictures on the side of the screen. Pick up one picture from each side (both hands) and drag them into the circle. | Bimanual upper limb coordination  Memory/cognition  Visual perception | 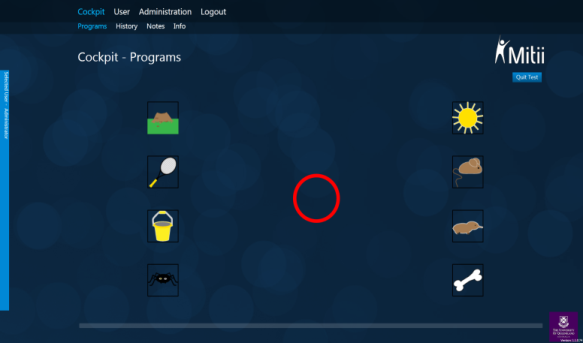 |
| Figure Ground | Impaired hand | Match the small piece of the larger picture | Down the bottom of the screen is a small part of a larger picture. Pick up the part at the bottom with your hand and then drag and hold it where it matches on the big background picture. | Upper limb movement  Visual perception | 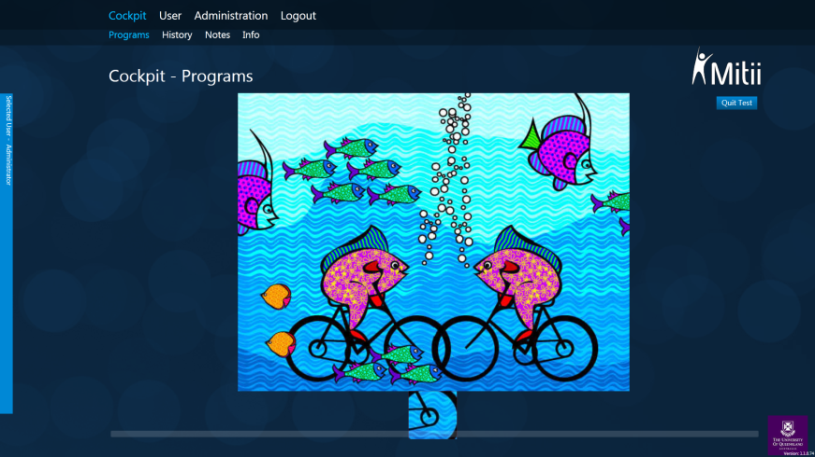 |
| Spatial Relations | Impaired hand | Select which picture does not match | Hold your hand over the picture which does not match (e.g. the first rabbit has both ears up). | Upper limb movement  Visual perception | 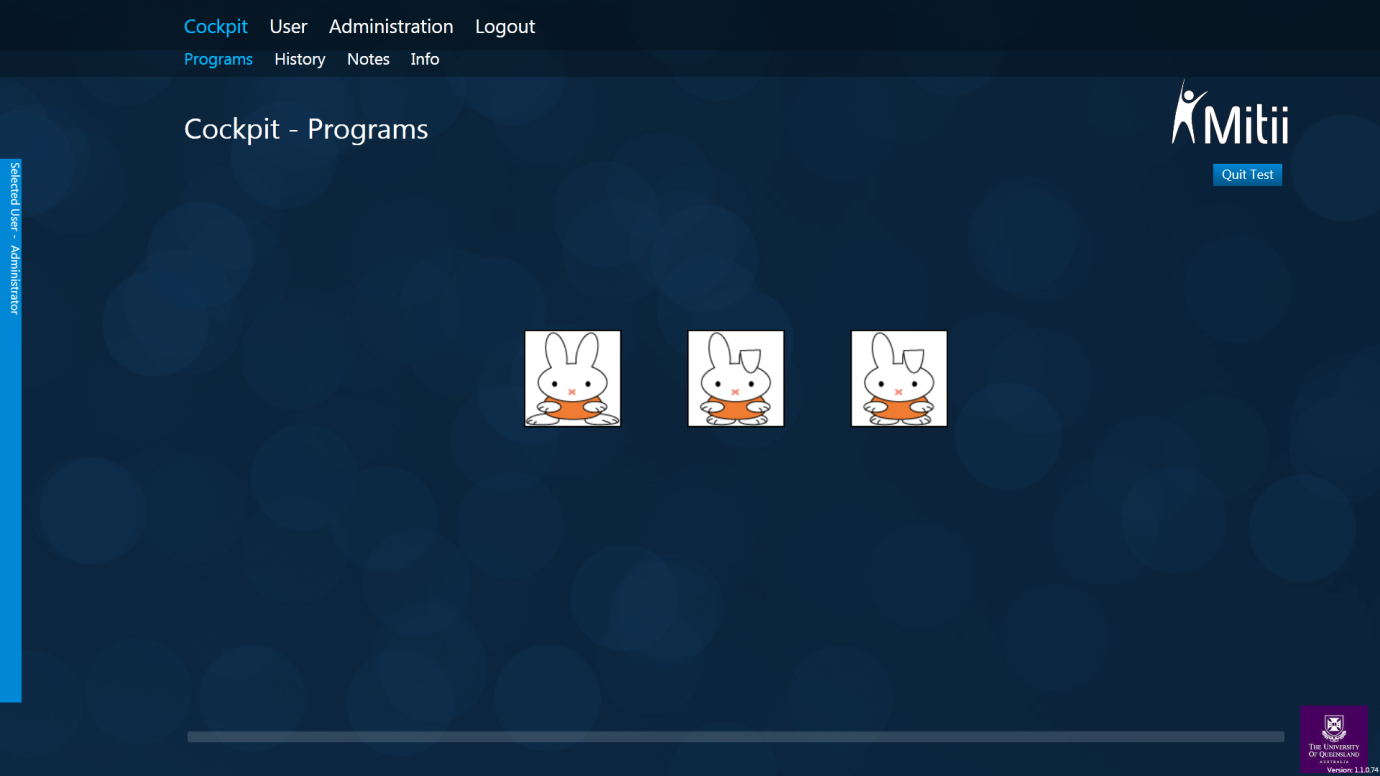 |
| Visual Closure | Impaired hand | Select which part-drawn or silhouette picture matches | *Type 1:*  A series of part-drawn pictures is shown.  Drag the part-drawn picture or the one that matches to the fully drawn picture underneath.  *Type 2:*  A series of pictures is shown.  Drag the picture that matches the silhouette picture. | Upper limb movement  Visual perception | 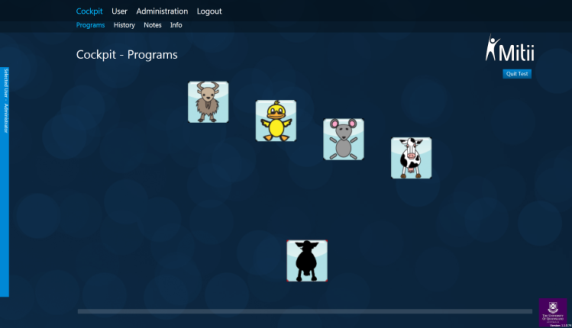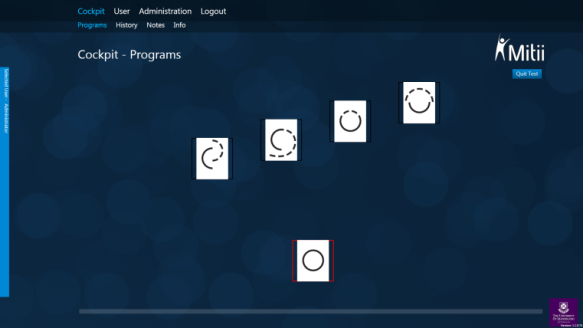 |
| UFO | Head/ Impaired hand | Steer the UFO through the tunnel | Squat or move your hand up and down to make sure the UFO does not hit the walls. | Head: lower limb strength, balance  Impaired hand: upper limb movement | 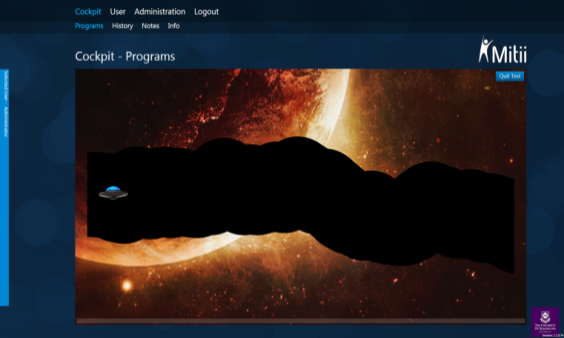 |
| Balloon mathematics | Impaired hand | Solve the mathematics problem | A maths question will be shown. Select the pin with your impaired hand (you may need to squat down) and drag it to the balloon with the correct answer to solve the maths problem. | Upper limb movement  Memory/cognition  Visual perception | 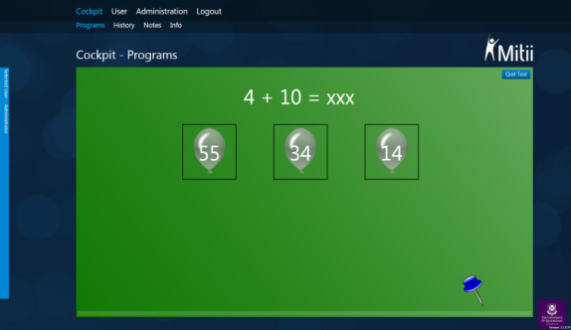 |
| Get up/Get Down | Head | Let’s get moving! Squat/jump/  Lunge etc. to get the object into the target | Try to fill up the glass of water, raise the temperature of the thermometer, count up to 10 or blow up the balloon until it bursts by moving (e.g. sit-to-stand, lunges, front/lateral step-ups on a block). | Lower limb strength  Balance | 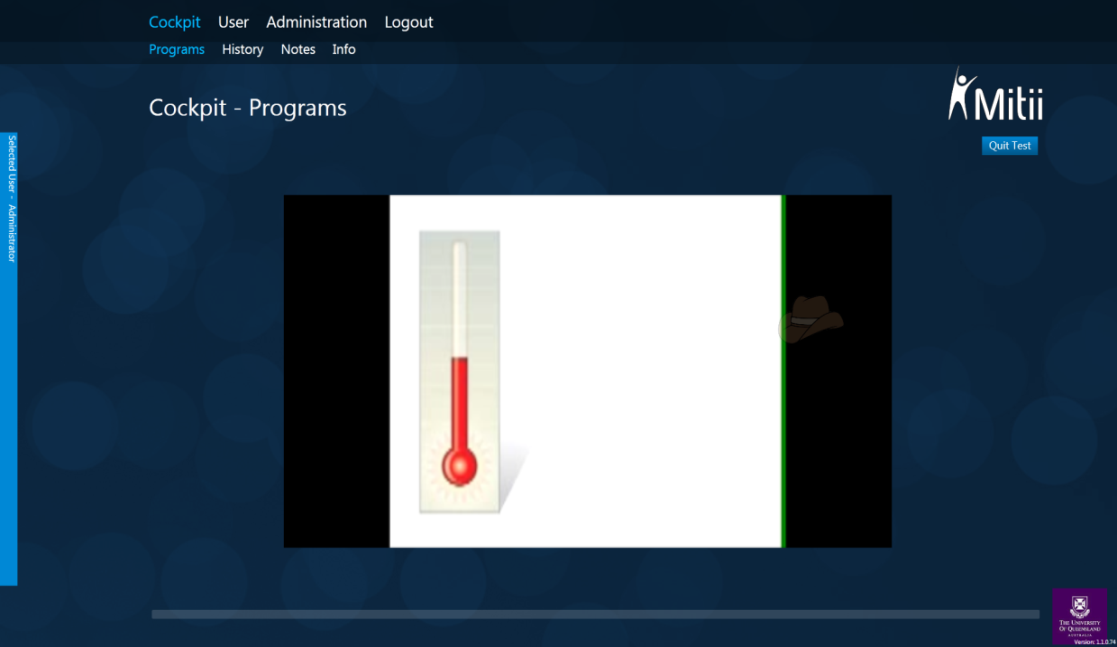 |
| Memory | Impaired hand | Memorising the order of pictures | Look at a series of pictures in red boxes.  These pictures then disappear and you must memorise them in the order in which they were shown. Hold your hand over picture to select the order. | Upper limb movement  Memory/cognition  Visual perception | 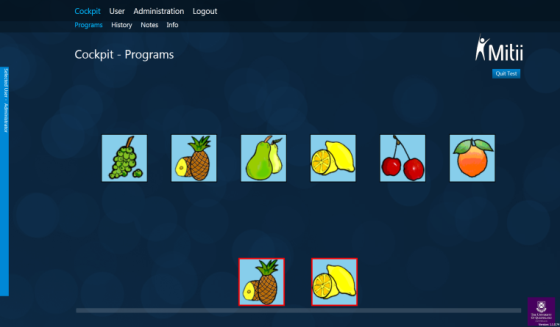 |
| Figure Builder (2-hand exercise) | Both hands | Construct the picture from smaller pieces falling down the side. Use your left hand for the left side and right hand for the right side. | The full picture is in the middle of screen.  Small pieces of this and other pictures are falling down the side.  Reach and drag the piece that matches using your hand to build the picture image from bottom to top. | Upper limb movement  Memory/cognition  Visual perception | 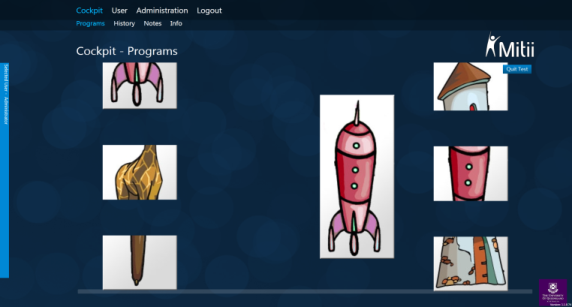 |
| Flight Simulator | Head | Keep the plane upright | Keep the middle of the plane as upright as possible while the wind tries to blow you off course. | Balance | 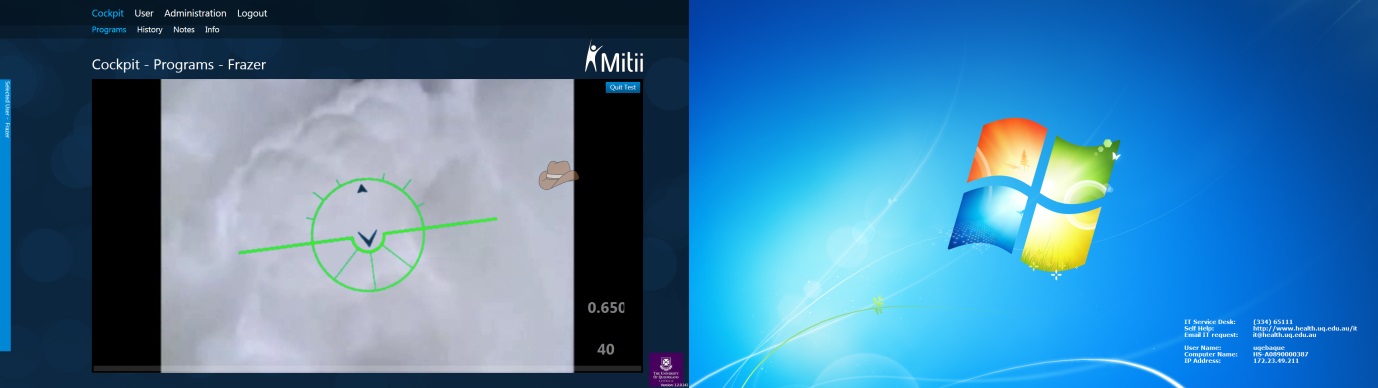 |
| Follow | Head/  Impaired Hand | Keep the man’s hands up | Keep the pistol close to the cowboy’s right side so his hands stay up. | Head: lower limb strength, balance  Impaired hand: upper limb movement | 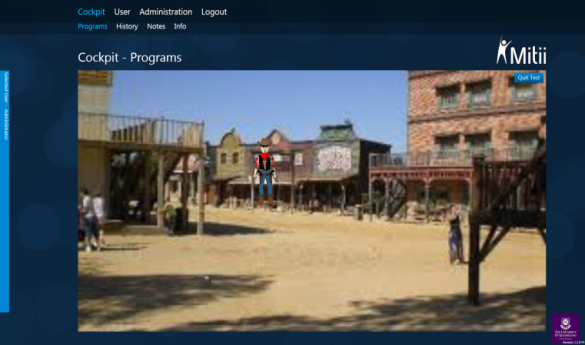 |
| Follow the leader | Head, arms and legs | Follow a sequence of movements | Follow what the leader is doing on the left hand side of the screen. You can see what you are doing on the right. The leader may ask you perform physical activities such as star-jumps, frog jumps, burpees or scissor jumps. | Lower limb strength  Balance  Upper and lower limb coordination | 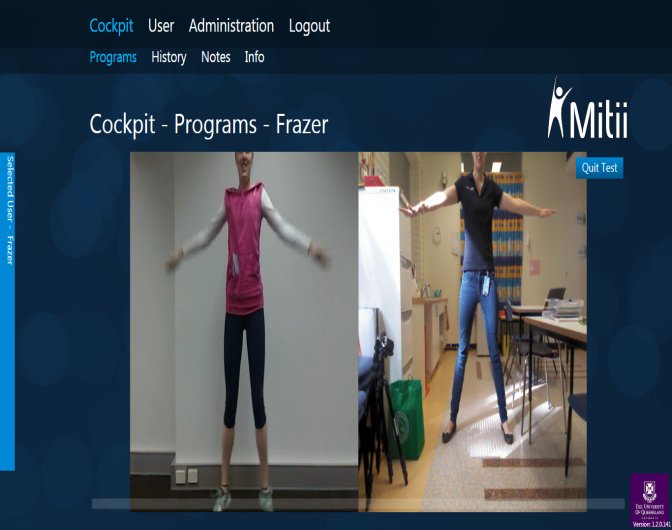 |
| Additional demands | Adding weights to upper and lower limbs to complete activities and/or wobble disc underneath feet to challenge balance | | | | |
